# Persuasive System Design Principles and Behavior Change Techniques to Stimulate Motivation and Adherence in Electronic Health Interventions to Support Weight Loss Maintenance: Scoping Review

**DOI:** 10.2196/14265

**Published:** 2019-06-21

**Authors:** Rikke Aune Asbjørnsen, Mirjam Lien Smedsrød, Lise Solberg Nes, Jobke Wentzel, Cecilie Varsi, Jøran Hjelmesæth, Julia EWC van Gemert-Pijnen

**Affiliations:** 1 Center for eHealth and Wellbeing Research Department of Psychology, Health, and Technology University of Twente Enschede Netherlands; 2 Research and Innovation Department Vestfold Hospital Trust Tønsberg Norway; 3 Center for Shared Decision Making and Collaborative Care Research Division of Medicine Oslo University Hospital Oslo Norway; 4 Norwegian Regional Advisory Unit on Patient Education Sørlandet Hospital Trust Kristiansand Norway; 5 Institute of Clinical Medicine Faculty of Medicine University of Oslo Oslo Norway; 6 Department of Psychiatry & Psychology Mayo Clinic Rochester, MN United States; 7 Saxion University of Applied Sciences Deventer Netherlands; 8 Morbid Obesity Center Vestfold Hospital Trust Tønsberg Norway; 9 Department of Endocrinology, Morbid Obesity, and Preventive Medicine Institute of Clinical Medicine University of Oslo Oslo Norway; 10 University Medical Center Groningen Groningen Netherlands; 11 University of Waterloo Waterloo, ON Canada

**Keywords:** eHealth, weight loss maintenance, weight loss, behavior change, persuasive technology, review, motivation, adherence

## Abstract

**Background:**

Maintaining weight after weight loss is a major health challenge, and eHealth (electronic health) solutions may be a way to meet this challenge. Application of behavior change techniques (BCTs) and persuasive system design (PSD) principles in eHealth development may contribute to the design of technologies that positively influence behavior and motivation to support the sustainable health behavior change needed.

**Objective:**

This review aimed to identify BCTs and PSD principles applied in eHealth interventions to support weight loss and weight loss maintenance, as well as techniques and principles applied to stimulate *motivation* and *adherence* for long-term weight loss maintenance.

**Methods:**

A systematic literature search was conducted in PsycINFO, Ovid MEDLINE (including PubMed), EMBASE, Scopus, Web of Science, and AMED, from January 1, 2007 to June 30, 2018. Arksey and O’Malley’s scoping review methodology was applied. Publications on eHealth interventions were included if focusing on weight loss or weight loss maintenance, in combination with motivation or adherence and behavior change.

**Results:**

The search identified 317 publications, of which 45 met the inclusion criteria. Of the 45 publications, 11 (24%) focused on weight loss maintenance, and 34 (76%) focused on weight loss. Mobile phones were the most frequently used technology (28/45, 62%). Frequently used wearables were activity trackers (14/45, 31%), as well as other monitoring technologies such as wireless or digital scales (8/45, 18%). All included publications were anchored in behavior change theories. *Feedback and monitoring* and *goals and planning* were core behavior change technique clusters applied in the majority of included publications. *Social support* and *associations* through prompts and cues to support and maintain new habits were more frequently used in weight loss maintenance than weight loss interventions. In both types of interventions, frequently applied persuasive principles were *self-monitoring*, *goal setting*, and *feedback*. *Tailoring*, *reminders*, *personalization,* and *rewards* were additional principles frequently applied in weight loss maintenance interventions. Results did not reveal an *ideal* combination of techniques or principles to stimulate motivation, adherence, and weight loss maintenance. However, the most frequently mentioned individual techniques and principles applied to stimulate motivation were, *personalization*, *simulation*, *praise,* and *feedback*, whereas *associations* were frequently mentioned to stimulate adherence. eHealth interventions that found significant effects for weight loss maintenance all applied *self-monitoring*, *feedback*, *goal setting*, and *shaping knowledge*, combined with a human *social support* component to support healthy behaviors.

**Conclusions:**

To our knowledge, this is the first review examining key BCTs and PSD principles applied in *weight loss maintenance* interventions compared with those of *weight loss* interventions. This review identified several techniques and principles applied to stimulate motivation and adherence. Future research should aim to examine which eHealth design combinations can be the most effective in support of long-term behavior change and weight loss maintenance.

## Introduction

### The Weight Loss Maintenance Challenge

Obesity is a rapidly increasing public health problem, with more than 600 million people with obesity (body mass index [BMI] ≥30 kg/m²) worldwide [1,2]. One of the main challenges related to obesity is that although many people manage to lose weight, only 1 of 4 people manages to maintain the lost weight in the long term [3]. With several mechanisms interacting (eg, biological, environmental, behavioral, and cognitive) to make weight regain following weight loss common [4,5], novel ways to counterbalance these mechanisms are needed [6,7]. The significant weight loss maintenance challenge calls for the examination of new technologies and solutions in future studies of long-term weight control [6,8,9].

### Electronic Health Design for Sustainable Behavior Change

eHealth (electronic health) is a term often used to define the blending of digital technologies, health, and health services and can be delivered through information and communication technologies [10-12]. Although evidence is sparse regarding the impact of eHealth on health and health care, research indicates that eHealth can support health care delivery by providing greater efficiency, lead to better health outcomes, and lower health service costs [10,13-15]. eHealth technologies are also increasingly used to support a healthier life, improved well-being, and creation of new health behaviors [16-19] and have the potential to support the challenging behavior changes needed to sustain long-term weight loss maintenance [4,20].

Behavior change interventions are usually complex and may include many interacting components or techniques [21,22]. Behavior change techniques (BCTs) are observable and active intervention components aiming to regulate behavior (eg, goal setting, self-monitoring, and feedback) [21,23]. However, health behavior change requires motivation and persistence, and persuasive design [24] also appears to play an important role in this setting. Persuasive design of services or technologies focuses on influencing human behavior in a positive way. As such, persuasive system design (PSD) principles can be applied in eHealth design to match user profiles, motivate users to engage in self-management, and trigger health behavior change [16,24-26]. Several behavior change theories, BCTs, and PSD principles can be involved in an eHealth intervention [27], alone or in combination.

To date, there is limited knowledge about how behavior change interventions and design of technologies and services can impact behavior and motivation in support of sustainable health behavior change [25,28,29]. eHealth is often described as a *black box*, as knowledge is limited about its internal structure and how the use of various components of the technology can contribute to healthier lifestyles and improved health outcomes [11,22,30,31]. Finding the right mix of technological features to stimulate the motivation and adherence needed to support long-term weight loss maintenance is, therefore, still a conundrum [7]. Little is also known about how BCTs and PSD principles can be used in eHealth interventions to support long-term weight loss maintenance [8,32]. The application of the most effective BCTs and PSD principles, at the right time and in the best combination, could therefore be of essence to support motivation and adherence in the pursuit of sustainable weight loss maintenance [19,33-35].

**The Goal of This Review**

The overall goal of this review was to provide insight into the design of eHealth interventions aiming to support behavior change for long-term weight loss maintenance in adult people with obesity. This review identified BCTs and PSD principles to stimulate motivation and adherence in eHealth interventions built to support weight loss maintenance.

Research questions for this review are as follows: in eHealth interventions, (1) how are *motivation* and *adherence* defined and measured? (2) Are *motivation* and *adherence* linked to weight loss and weight loss maintenance? (3) What can be determined from behavior change theories, BCTs, and PSD principles used in weight loss and weight loss maintenance interventions? (4) Which behavior change theories, BCTs, and PSD principles have been used to stimulate motivation and adherence in eHealth weight loss maintenance interventions, and in what combination? (5) What are the reported effects (ie, weight outcomes) in eHealth weight loss maintenance interventions?

## Methods

### Scoping Review Methodology

A scoping review methodology was considered suitable for mapping literature on BCTs and PSD principles, as this is an emerging topic where evidence is scarce and key concepts and gaps in existing research should be identified [36,37]. This scoping review applied the methodology by Arksey and O’Malley [36], with the following steps [37]: (1) identify the research questions; (2) identify relevant studies; (3) study selection; (4) chart the data; (5) collate, summarize, and report the results; and (6) consultation. To enhance the scoping study methodology, additional recommendations [37] were followed: (1) 2 independent researchers reviewed all full-text publications, and (2) the research group developed and continuously updated the data extraction form during the extraction process.

As research on eHealth interventions targeting weight loss maintenance is still in its infancy, eHealth interventions targeting weight loss were also examined to best identify weight loss maintenance–related factors. Research questions 1, 2, and 3 entailed broad scopes. The scope was then further narrowed in research questions 4 and 5, focusing on weight loss maintenance interventions and the BCTs and PSD principles applied to stimulate motivation and adherence, as well as any effects (ie, weight outcomes) related to weight loss maintenance.

This review applied Michie’s Behavior Change Taxonomy [21] developed to meet the need for standardized reporting on development and content of complex behavior interventions. Michie’s Cross-Domain Taxonomy consists of 93 distinct BCTs divided into 16 clusters, independent of any specific theory. Multimedia Appendix 1 provides detailed information about the BCT clusters. Similarly, the PSD model by Oinas-Kukkonen [16], building on previous research by Fogg [26], was used as a framework to identify persuasive principles applied in the included interventions. Multimedia Appendix 2 provides information about the 4 PSD categories: *primary task support*, *dialog support*, *system credibility support*, and *social support*, as well as operationalization of the individual principles [16]. For the purpose of this review, adherence to a technology was defined as *use as intended or desired* by the authors or developers of an intervention [19], whereas motivation was defined as *a reason for doing something* [38].

### Search Strategy

A systematic literature search to cover behavioral, technical, and clinical research aspects was conducted in the following databases: PsycINFO, Ovid MEDLINE (including PubMed), EMBASE, Scopus, Web of Science, and AMED. As digital technologies are advancing and developing fast, more recent evidence (ie, since 2007) was considered to be the most relevant and interesting. Publications during the period from January 1, 2007 to June 30, 2018, were therefore included. The terms *weight loss* and *weight loss maintenance* were used, in combination with a variety of the term *eHealth interventions* and the terms *motivation*, *adherence*, and/or *behavior change*. This search strategy was created and applied in close collaboration with librarians and domain experts (Multimedia Appendix 3).

### Eligibility Criteria

Publications in English, clearly describing an eHealth intervention focusing on weight loss maintenance or weight loss, were included for assessment when containing persuasive design, behavior change theories, and techniques or when mentioning motivation and/or adherence. The target population for this review was people with overweight (ie, BMI 25-29.9 kg/m^2^) and/or obesity (ie, BMI ≥30 kg/m^2^). Multimedia Appendix 4 gives a complete overview of the inclusion and exclusion criteria.

### Data Collection and Analysis

A data charting form containing general as well as specific study characteristics was created in Microsoft Excel by the research team. The characteristics were extracted using elements from the CONSORT-eHealth checklist [39], focusing on characteristics about the interventions and technologies in the included publications. Michie’s Cross-Behavior Change Taxonomy [21] guided the extraction process to identify and group specific information about BCTs used. For the purpose of this review, representation of the 16 clusters as indicated in Multimedia Appendix 1 was applied, rather than presenting a detailed representation of up to 93 distinct techniques. Supplementary information about definitions, including specific examples, was reviewed [40], and behavior change theories mentioned or described were recorded. Persuasive principles were extracted from the included publications using the PSD model [16] presented in Multimedia Appendix 2. PSD principles were coded when executed by or through the technology. Due to lack of reporting on the system credibility category, this category was not part of the analysis. *Goal setting*, *feedback*, and *social support* were added to the model as separate persuasive principles, as they could not always easily be linked to specific design elements in the PSD model.

Two researchers (RA and MS) independently coded and categorized the identified BCTs and PSD principles, using the presented frameworks. A third researcher (CV) validated 11% (5/45) of the included interventions. The first and second author also recorded additional information on motivation and adherence when mentioned, including how motivation/adherence was defined, stimulated, and measured. Data on effect (ie, weight outcomes) were recorded when reported, including when weight was self-reported or measured by the researcher or coach/clinician. The research team also extracted relevant information (eg, intervention components, BCTs, and PSD principles) from the incorporated interventions, including illustrations, figures, tables, and additional websites when referred to in the publications. To enhance and support the relevance of the review, clinicians and researchers specialized in the fields of weight loss/maintenance, health psychology, and eHealth were consulted regarding methodological approach, relevance, and current state of the evidence.

### Weight Loss and Weight Loss Maintenance

Weight loss BCTs do not necessarily equal weight loss maintenance BCTs. Long-term maintenance of lost weight is challenging, and there is a call for interventions evaluating novel methods to improve the maintenance of lost weight [8,41]. To meet this call and the overall goal of this review (ie, supporting long-term weight loss maintenance), the first part of the Results section focuses on weight loss and weight loss maintenance, whereas the rest of the results section focuses solely on weight loss maintenance interventions.

## Results

### Study Selection

**Figure 1:**
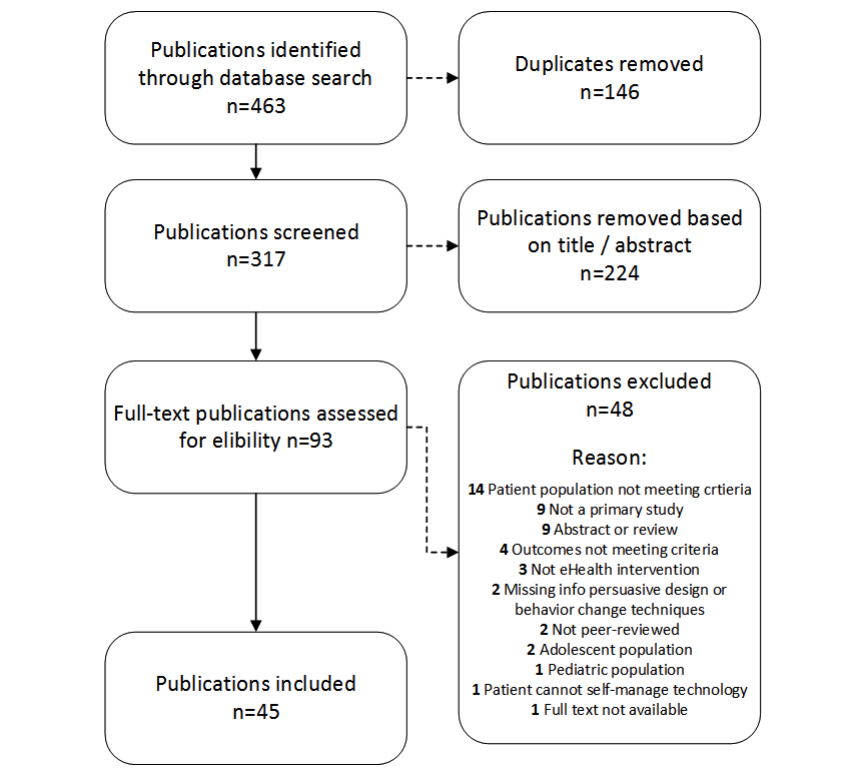
Flow diagram of study selection process. n=the actual number of publications. eHealth: electronic health.

The search revealed 463 publications. Following removal of 146 duplicates, the remaining 317 titles and abstracts were screened for eligibility by 2 researchers (RA and either MS, CV or FS) using the Covidence software program [42]. After removing 224 publications not meeting eligibility based on title/abstracts, the remaining 93 full-text publications were reviewed by the first (RA) and second (MS) authors. The authors RA and MS discussed differences and disagreements until consensus was reached. If consensus could not be reached, consultation was sought from a third researcher (FS)*.* After the full-text screening process, 45 publications remained to be assessed for further analyses (Figure 1).

### General Characteristics of Included Publications

**Figure 2:**
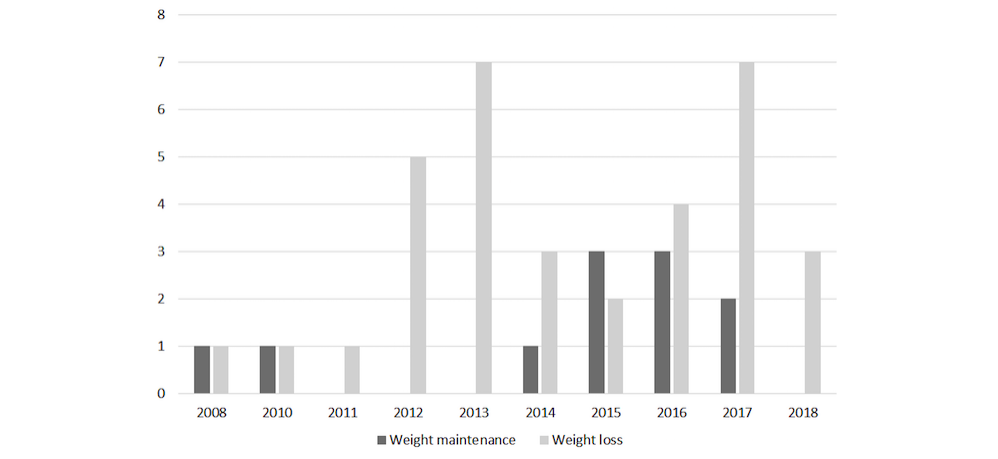
The number of included publications per year categorized by the aim of the electronic health intervention.

All 45 publications included in this review were published in 2008 or later (Figure 2). First authors were mainly affiliated in the United States (25/45, 56%), the United Kingdom (5/45, 11%), and Australia (3/45, 7%); see Table 1.

**Table 1:** Country of affiliation for the first authors of all included publications (N=45).

Country of origin	Included publications, n (%^a^)
United States	25 (56)
United Kingdom	5 (11)
Australia	3 (7)
Canada	2 (4)
The Netherlands	2 (4)
Finland	1 (2)
Germany	1 (2)
Italy	1 (2)
Scotland	1 (2)
Saudi Arabia	1 (2)
Spain	1 (2)
Romania	1 (2)
Qatar	1 (2)

^a^Percentages do not total 100% due to rounding errors.

Of the 45 included interventions, 34 (76%) targeted weight loss, and 11 (24%) targeted weight loss maintenance. Of 11 weight loss maintenance interventions, 5 (45%) had an initial weight loss phase. The most frequently described study design was randomized controlled trials for the weight loss (15/45, 33%) and weight loss maintenance (4/45, 9%) interventions alike. Although several of the included interventions evaluated effects (ie, weight outcome; 26/45, 58%), others described only the design of the intervention (6/45, 13%) or a protocol (7/45, 16%). Most interventions targeted people with overweight (24/45, 53%) or included both overweight and obesity (15/45, 33%). People with obesity (ie, BMI ≥30 kg/m^2^) were the sole target population in only 4 weight loss interventions and 2 weight loss maintenance interventions. Average duration of interventions was 24 weeks (range 4-104; median 13 weeks) for the weight loss interventions, and 27 weeks (range 12-52; median 26 weeks) for the weight loss maintenance interventions. User involvement during the technology development process was only described by 8 weight loss and 4 weight loss maintenance interventions. The users were usually only mentioned when involved in part of the development process (eg, identifying needs, content development, and usability testing). Two publications were included despite describing the same intervention, as they focused on different aspects of the intervention [43,44]. Multimedia Appendix 5 provides an overview of the included interventions including title, authors, publication year, country of origin, design, objectives, participants, aim and type of technology, intervention duration, and whether blended care was part of the intervention or not.

### Technology Characteristics

In the 45 included publications, mobile phones were the most frequently used technology (28/45, 62%), followed by Web-based solutions through computers (15/45, 33%), or a combination of computer and mobile phone (eg, for feedback or reminders through text messages; 6/45, 13%). Monitoring technologies used were activity trackers or step counters (14/45, 31%), wireless or digital weight scales (8/45, 18%), and glucose (2/45, 4%) or blood pressure (1/45, 2%) monitors, often combined with manually recorded self-monitoring data. Some technologies included game-based elements (3/45, 7%) [45-47], a virtual world with avatars [48] or a virtual coach [49], and other tools to enhance self-monitoring (eg, automated calculations of energy intake, expenditure, and energy balance). Several eHealth weight loss interventions (20/45, 44%) and weight loss maintenance interventions (7/45, 16%) had a blended care approach, including the combination of various formats of human coaching and/or face-to-face services from experts. Some eHealth interventions also integrated peer forums or social media groups (7/45, 16%) or included social support through a buddy or helper (eg, family, friend, colleague; 3/45, 7%).

The typical eHealth weight loss maintenance intervention was supported by mobile phone technology (9/11, 82%), in combination with an activity tracker or step counter (3/11, 27%) and/or wireless scale (3/11, 27%) [47,50-52]. The technology usually supported 2-way communication with a peer, dietitian, or coach [50,53-56] and provided automated, tailored feedback based on progress data [50-53,55,57,58]. The technology in these weight loss maintenance interventions usually aimed to support creation of healthy habits [51-54]; educational resources and information [51,53,54,59]; daily or weekly monitoring tools for weight, diet, activity (eg, number of steps) [47,50-52,55]; well-being (eg, mood, stress, and good days/bad days) [47,51,53]; and/or plans or strategies for individual action and coping [47,54,57].

### Motivation Defined, Measured, and Linked to Weight Loss and Weight Loss Maintenance

Only 2 of the 45 included publications provided a *definition* of motivation [43,44], and these 2 publications originated from the same intervention. By referring to self-determination theory, the publications distinguished between autonomous motivation (a measure of a person’s *internal or personal reasons for change*) or controlled motivation (a measure of *extrinsic reasons or external pressure to change*) [44].

Motivation was *measured* in various ways by self-reported measures and questionnaires as presented in Table 2.

Motivation was *evaluated* (ie, *link to weight loss/maintenance*) in 7 of 35 weight loss intervention studies [43,44,49,60,63-65], but in none of the included weight loss maintenance interventions (n=11) as indicated in Multimedia Appendix 6. Of the 7 weight loss studies that evaluated motivation, 1 found high levels of controlled motivation at baseline to produce significantly greater weight loss in the motivation-enhanced intervention (ie, specific components were used to enhance autonomous motivation) compared with the standard intervention [43]. The motivation-enhanced group used the website more often than the control group, and the number of visits was associated with weight loss [43]. To increase autonomous motivation, principles of motivational interviewing [66] together with goal setting and journaling (eg, writing about the future when weight loss goals are achieved) and blended formats, such as face-to-face sessions, were also added to the Web-based weight loss program to improve autonomous motivation [43].

Another study showed the level of autonomous motivation after 4 weeks to be predictive of self-monitoring of adherence and weight loss at 16-week postbaseline [44]. For participants who reached 5% weight loss, autonomous motivation increased and remained higher than for those not reaching this clinically meaningful weight loss [44]. A third study suggested that diet-focused constructs were particularly important when developing weight loss interventions for men [60] because changes in diet-related autonomous motivation were linked to weight loss. In that study, the intervention group achieved greater weight loss than the control group [60].

The fourth study evaluating motivation found motivational orientation (eg, promotion focused or prevention focused) to be a predictor of behavior change when trying to lose weight, and framing messages with people’s motivational orientation was considered preferable to, for example, informational and prescriptive messages in terms of behavior change [63]. As high as 72% of the participants in another study found text messages received biweekly to be motivational [64], and 79% reported text messages to be helpful in performing healthy eating and exercise behaviors [64]. The sixth study evaluating motivation found that delivery of remote daily real-time feedback messages tailored to diary entries could enhance motivation, producing greater reductions in energy and saturated fat consumptions [67]. In the final study, 58% of the participants agreed that a virtual coach motivated them to become more active [49], suggesting that meetings with a virtual coach could be beneficial in maintaining activity level. However, no significant changes in step count were found in the intervention versus the control groups [49].

**Table 2:** Measurements of motivation.

Methods for measuring motivation	Reasons to measure motivation	Publications^a^	P^b^/I^c^
Treatment Self-Regulation Questionnaire	To assess autonomous versus controlled motivation for self-regulation, weight loss, healthy eating, and continued exercise	WL9^d^ [60]; WL25 [61]; WL30 [43]; WL31[44];	I; P; I; I
Behavioral Regulation in Physical Exercise and Eating Habit Questionnaire	To assess treatment moderators (here, intrinsic motivation) and measure stages of the self-determination continuum, a motivation factor	WL4 [62]	P
Consideration of Future Consequences	To characterize motivational orientation and measure participants’ focus on distal versus proximal consequences/outcomes of behaviors	WL8 [63]	I
Behavioral Inhibition and Behavioral Activation Scales	To identify participants’ motivational orientation, either predominantly promotion focused (gain focus) or predominantly prevention focused (loss focus)	WL8 [63]	I
The Diet and Exercise Self-Efficacy Questionnaires	To assess self-efficacy to make and maintain diet and exercise behavior changes	WL8 [63]	I
The University of Rhode Island Change Assessment scale	On the basis of the transtheoretical (stages-of-change) model, to assess where an individual exists along a 5-phase continuum from precontemplation to contemplation, preparation, action, and maintenance	WL8 [63]	I
Online survey	To map to what extent text messages were experienced as motivational	WL5 [64]	I
Online self-reporting	To rate their motivation and confidence to continue their weight next week	WM2^e^ [51]	P
Online self-reporting/feedback	To set the level of participant motivation	WL23 [65]	I

^a^Multimedia Appendix 5 provides an overview of the publications.

^b^P: protocol.

^c^I: intervention.

^d^WL: weight loss.

^e^WM: weight loss maintenance.

### Adherence Defined, Measured, and Linked to Weight Loss and Weight Loss Maintenance

Of the 45 included publications in this review, 6 weight loss interventions measuring adherence provided a *definition* of the adherence concept or related terms such as usage and compliance [64,68-72]. These publications defined or operationalized adherence based on either self-monitoring or electronic entry of food and exercise records [68]. Low usage was defined as having no food records and high usage as having 1 food record on a randomly selected day of the sampled week [68] or as recording of 50% or more of prescribed daily calorie intake goal [69]. Some publications also defined adherence as responsiveness to text messages or health challenges [64,71,72] or looked at various aspects of adherence such as behavioral adherence (ie, attendance to counseling sessions) [68] and dietary adherence (ie, self-monitoring related to dietary goals) [68,70]. Other publications defined adherence as program compliance to habits and workout [71] or consistency to self-monitoring [73].

Regarding *measurement* of adherence, as the main scope of this review was weight loss maintenance, the results related to short-term weight loss interventions (≤6 months) measuring adherence are not reported in this review [18,43,44,49,69,70,73-77]. Moreover, 4 of the included long-term weight loss interventions (>6 months) measured adherence [68,71,72,78]. For weight loss maintenance, adherence was measured in 3 of the interventions [50,52,59], and the duration for 2 of these interventions was more than 6 months (Multimedia Appendix 6). The 4 long-term weight loss interventions measured various aspects of adherence, including self-monitoring data related to diet and physical activity [68], compliance to the Web-based program, daily habits and exercise [71], website usage by the number of self-tracking entries [78], or the total percentage of text messages that a participant responded to [72]. The 3 weight loss maintenance interventions measured adherence or engagement in relation to the coaching program through evaluating frequency of submitting self-monitoring data to their coach [52], the number of delivered text messages replied to by the participants [59], and by participants’ self-monitoring adherence through frequency of weigh-ins and use of activity tracking [50].

Adherence to technology was defined as *use as intended or desired* in this review [19]. Intended usage was reported in the 4 weight loss interventions (>6 months) and in all 3 weight loss maintenance interventions. Intended use was most frequently described as 1 time per day or more (≥1 per day).

#### Links to Weight Loss and Weight Loss Maintenance

Although actual technology usage was not evaluated in the 4 weight loss interventions measuring adherence, and significant results were sparsely reported, some interventions measured certain aspects of the technology features and linked these to weight loss. However, 1 intervention showed that participants with a high usage of self-tracking entries initially lost greater amounts of weight than participants with low usage [68]. Other interventions reported that compliance to the Web-based program, daily habits, and exercise was also found to be a significant predictor of weight loss [71], and that participants with greater adherence to text messages lost more weight [72].

The 3 weight loss maintenance interventions measured adherence, showing significant effects (ie, weight loss maintenance) at 12 weeks postbaseline, 12 months postintervention, and 24 months postbaseline [50,52,59]. It should be noted that the methods applied and/or results reported only provided results related to intervention engagement and technology or intervention features, not related to the actual use of the technology [50,52,59] (Multimedia Appendix 6).

### Behavior Change Theories and Behavior Change Techniques in Weight Loss and Weight Loss Maintenance Interventions

#### Behavior Change Theories Applied in Weight Loss and Weight Loss Maintenance Interventions

All 45 included publications were theoretically anchored in behavior change theories. In approximately two-thirds of the interventions, specific behavioral change theories were mentioned as applied. Interventions that did not specify behavior change theories referred to behavioral strategies or techniques as crucial factors for behavior change [18,50,52,55,58,62, 64,67-70,75,78-82]. Multimedia Appendix 7 shows an overview of the behavior change theories specified as applied in the weight loss and weight loss maintenance interventions.

#### Behavior change technique clusters applied in weight loss and weight loss maintenance interventions

Analysis of all included publications (N=45) identified 15 of the 16 BCT clusters specified in Michie’s taxonomy [21] (Table 3).

The *goal and planning* and *feedback and monitoring* clusters were referred to as core self-regulation techniques for behavior change and weight outcomes in several of the publications [51,52,54,74,77]. These cluster techniques were also applied in most of the weight loss *and* weight loss maintenance interventions. Techniques contributing to the cluster *shaping knowledge* were present in 82% of the interventions (weight loss and weight loss maintenance). This cluster included providing relevant information on diet, physical activity, and how to change behavior, advice on how to perform a desired behavior, or advice to keep a record on social situations, emotions, or cognitions that typically occur before temptations (eg, snacking) [47,50,52,54,56,58,59]. *Social support* was a more frequently used technique in weight loss maintenance (91%) than in weight loss (68%) interventions, enabled with as well as without technology. *Social support* was typically provided through e-coaching and social reinforcement from professionals or peers [45,51-53,58,80], with encouragement and counseling on performed behavior [52,57].

**Table 3:** Behavior change cluster of techniques according to Michie’s taxonomy.

Cluster labels	WM^a^ (n=11), n (%)	WL^b^ (n=34), n (%)	All (N=45), n (%)
Scheduled consequences	1 (9)	2 (6)	3 (7)
Reward and threat	3 (27)	4 (12)	7 (16)
Repetition and substitution	8 (73)	24 (71)	32 (71)
Antecedents	4 (36)	8 (24)	12 (27)
Associations	8 (73)	15 (44)	23 (51)
Covert learning	0 (0)	0 (0)	0 (0)
Natural consequences	3 (27)	7 (21)	10 (22)
Feedback and monitoring	11 (100)	34 (100)	45 (100)
Goals and planning	11 (100)	33 (97)	44 (98)
Social support	10 (91)	23 (68)	33 (73)
Comparison of behavior	0 (0)	9 (26)	9 (20)
Self-belief	2 (18)	6 (18)	8 (18)
Comparison of outcomes	2 (18)	4 (12)	6 (13)
Identity	1 (9)	4 (12)	5 (11)
Shaping knowledge	9 (82)	28 (82)	37 (82)
Regulations	3 (27)	3 (9)	6 (13)

^a^WM: weight loss maintenance interventions.

^b^WL: weight loss interventions (Multimedia Appendix 5 provides an overview of the publications).

Michie’s cluster *associations* were more frequently applied in weight loss maintenance (73%) than in weight loss (44%) interventions, and these techniques were often an environmental or social stimulus or reminders with the purpose of prompting a specific behavior [52,54,56,57]. The *comparison of behavior* cluster was only present in 26% of the weight loss interventions and not identified at all in the weight loss maintenance interventions [43-45,71,75,80,83,84].

#### Persuasive System Design Principles Applied in Weight Loss and Weight Loss Maintenance Interventions

An overview of the PSD principles applied by or through the technology in the included publications (N=45) is presented in Table 4. In the included interventions, the *primary task support* category from the PSD model [16], supporting users to do primary tasks, was applied most often (50%), followed by *dialog support* (35%) and *social support* (15%).

**Table 4:** Persuasive system design principles.

Persuasive principles		WM^a^ (n=11), n (%)	WL^b^ (n=34), n (%)	All (N=45), n (%)
**Primary task support**				
	Self-monitoring	11 (100)	30 (88)	41 (91)
	Tailoring	11 (100)	22 (65)	33 (73)
	Personalization	8 (73)	16 (47)	24 (53)
	Simulation	8 (73)	15 (44)	23 (51)
	Reduction	3 (27)	4 (12)	7 (16)
	Tunneling	3 (27)	5 (15)	8 (18)
	Rehearsal	2 (18)	5 (15)	7 (16)
**Dialog support**				
	Reminders	9 (82)	15 (44)	24 (53)
	Suggestions	7 (64)	20 (59)	27 (60)
	Reward	6 (55)	5 (15)	11 (24)
	Praise	4 (36)	13 (38)	17 (38)
	Social role	1 (9)	2 (6)	3 (7)
	Similarity	0 (0)	2 (6)	2 (4)
	Liking	0 (0)	1 (3)	1 (2)
**Social support**				
	Social comparison	2 (18)	8 (24)	10 (22)
	Social facilitation	2 (18)	3 (9)	5 (11)
	Social learning	1 (9)	7 (21)	8 (18)
	Cooperation	1 (9)	3 (9)	4 (9)
	Recognition	1 (9)	3 (9)	4 (9)
	Competition	0 (0)	4 (12)	4 (9)
	Normative influence	0 (0)	1 (3)	1 (2)
**Other**				
	Feedback	11 (100)	31 (91)	42 (93)
	Goal setting	10 (91)	27 (79)	37 (82)
	Social support	7 (64)	18 (53)	25 (56)

^a^WM: weight loss maintenance interventions.

^b^WL: weight loss interventions.

In the weight loss interventions, the most frequently applied persuasive principles were *feedback* (91%), *self-monitoring* (88%), *goal setting* (79%), *tailoring* (65%), and *suggestions* (59%). In the weight loss maintenance interventions, *feedback*, *self-monitoring,* and *tailoring* (all 100%) were the most frequently applied persuasive principles, followed by *goal setting* (91%) and *reminders* (82%). *Social support* as a PSD principle, usually 2-way communication with peers or a coach, was used to support continued behavior change [46,54,56,84] and was identified and applied almost as often in weight loss (53%) as in weight loss maintenance (64%) interventions. Frequently reported PSD principles in weight loss maintenance interventions compared with weight loss interventions were *personalization* (73% vs 47%), *simulation* (73% vs 44%), and *rewards* (55% vs 15%). *Competition*, on the other hand, was one of the persuasive principles not identified in any weight loss maintenance interventions, although identified in 12% of the weight loss interventions, often related to weight changes and/or activity targets [45,75,84,85]. Application of the *self-monitoring* principle, one of the most frequently applied principles in both types of interventions, was associated with user recording of weight and behaviors connected to diet and physical activity targets [51,53,57] and reception of automated, *tailored feedback* through text messages or visually by graphs, charts, bars, symbols (eg, traffic light, colors when entering a *danger zone*) [51], and dashboard [50] related to their progress. *Normative influence*, *similarity*, and *liking* were the persuasive principles least applied in both types of interventions (Table 4). An overview of PSD principles identified in the included weight loss and weight loss maintenance interventions can be found in Multimedia Appendix 8.

### Behavior Change Theories, Behavior Change Techniques, and Persuasive System Design Principles Used to Stimulate Motivation and Adherence in Electronic Health Weight Loss Maintenance Interventions

As seen in Tables 3 and 4, weight loss BCTs and PSD principles do not necessarily equal weight loss maintenance techniques. To meet the call for interventions evaluating novel methods to improve maintenance of lost weight [8] and meet the overall goal of this review, the next part of the Results section focuses solely on weight loss maintenance interventions.

### Behavior Change Theories and Techniques Used to Stimulate Motivation and Adherence in Electronic Health Weight Loss Maintenance Interventions

#### Behavior Change Theories

Of the 11 weight loss maintenance interventions included in this review, 7 explicitly mentioned which behavior change theories were used [47,51,53,54,56,57,59] but did not describe using these to stimulate motivation or adherence directly as indicated in Table 5. Of the publications that described reasons for applying the identified behavioral theories, the following were mentioned: (1) to support long-term behavior change by increasing the individual coping capacity [57], (2) to facilitate goal setting, monitoring, and feedback [51], (3) to adapt the text messages to participant’s readiness for change [59], (4) to support existing behavior change, and (5) to develop new self-management skills [56]. Motivational interviewing was added to 1 eHealth intervention [53] through physical consultation to enhance adherence.

#### Behavior Change Techniques

Table 5 shows that all 11 weight loss maintenance interventions used various BCTs to stimulate weight loss maintenance. Of 11 interventions, 9 applied BCT clusters to stimulate motivation and/or adherence. Analyses indicate that *feedback and monitoring* was the most frequently mentioned cluster to stimulate motivation (55%) [47,51,53,54,57,58]. Within this cluster, motivation was enhanced through encouraging and supporting usually by automated messages [47,51,53,57]. Face-to-face contact was also offered to enhance participants’ motivation to engage with internet-delivered elements [51]. *Monitoring* was also mentioned as a key strategy to achieve weight behavior change and weight control in several studies, and different BCTs were used to support manually and automated *monitoring* of goals and target behavior [47,50-54,56,57]. *Associations* were, as indicated in Table 5, applied most often to stimulate adherence through *prompts and cues* in the weight loss maintenance interventions (27%) [54,56,57].

In 1 study, 3 clusters of BCTs, *feedback and monitoring*, *goals and planning,* and *social support*, were applied to stimulate motivation [52]. Target behavior was emphasized to be reduced from great overall goals to *small steps*, which could be more easily reached by the participants [53]. Other studies showed *reward* as 1 of the clusters mentioned applied to stimulate motivation as well as adherence in support of weight loss maintenance by linking financial rewards to submitting self-monitoring records ($1–$10 per week) [52] or accomplishing behavioral goals (eg, by offering direct payments ($2.80) each day the participants weighed in and met the weight loss goals) [55]. Incentive *payouts* were also made contingent on a *dyadic partner performance* to stimulate adherence [52], meaning that members of the partner dyad had to email for 5 days or more about self-monitoring, and both partners had to maintain their weight loss to get the financial incentives. The cluster *shaping knowledge* was used to offer general suggestions and theory-based advice on how to maintain weight loss, not to stimulate motivation or adherence (eg, information and supportive tools on diet, physical activity, and behavior change) [50,51,54,56,59]. A serious game-based eHealth intervention also offered access to information while playing (eg, general information about dieting, research news, and fact sheets) [58].

**Table 5:** Included weight loss maintenance interventions specifying behavior change theories and behavior change technique (BCT) clusters.

Study ID^a^		WM^b^ 1 [57]	WM 2 [51]	WM 3 [52]	WM 4 [59]	WM 5 [55]	WM 6 [56]	WM 7^c^ [54]	WM 8 [53]	WM 9 [50]	WM 10 [47]	WM 11 [58]
**Behavior change theories mentioned used**												
	Social cognitive theory	—^d^	—	—	—	—	—	—	—	—	—	—
	Cognitive behavioral therapy, ABC model	✓^e^	—	—	—	—	—	—	—	—	✓	—
	Health action process approach model	✓	—	—	—	—	—	—	—	—	—	—
	The transtheoretical model	—	—	—	✓	—	✓	—	—	—	—	—
	Goal setting and action theories	—	—	—	—	—	—	✓	—	—	—	—
	Self-regulation theory	✓	✓	—	—	—	—	✓	—	—	—	—
	Regulatory fit theory	—	—	—	—	—	—	—	—	—	—	—
	Control theory	—	—	—	—	—	—	—	—	—	—	—
	Self-determination theory	—	—	—	—	—	—	—	—	—	—	—
	Social support theories	—	—	—	—	—	✓	—	—	—	—	—
	Motivational interviewing	—	—	—	—	—	✓	—	✓	—	—	—
	Stroebe’s theory on behavior change	—	—	—	—	—	—	—	—	—	—	—
	Conservation of resources theory	✓	—	—	—	—	—	—	—	—	—	—
	Michie’s Behavior Change Wheel framework	—	—	—	—	—	—	—	—	—	—	—
	Self-directed behavior change theory	—	—	—	—	—	✓	—	—	—	—	—
**Michie’s Behavior Change Taxonomy**												
	Scheduled consequences	—	—	—	—	—	✓	—	—	—	—	—
	Reward and threat	—	—	A^f^	—	M^g^ / A	—	—	—	—	—	—
	Repetition and substitution	✓	✓	—	✓	—	✓	✓	—	✓	✓	✓
	Antecedents	✓	✓	—	—	—	—	✓	—	—	✓	—
	Associations	A	✓	✓	—	✓	A	A	—	✓	✓	—
	Covert learning	—	—	—	—	—	—	—	—	—	—	—
	Natural consequences	✓	✓	—	—	—	—	—	—	—	—	—
	Feedback and monitoring	M	M	✓	✓	✓	✓	M	M	✓	M	M
	Goals and planning	✓	✓	✓	✓	✓	✓	✓	M	✓	✓	✓
	Social support	✓	M	✓	✓	—	✓	✓	M / A	✓	—	✓
	Comparison of behavior	—	—	—	—	—	—	—	—	—	✓	—
	Self-belief	✓	✓	—	—	—	—	—	—	—	—	—
	Comparison of outcomes	—	✓	—	—	—	—	✓	—	—	—	—
	Identity	—	✓	—	—	—	—	—	—	—	—	—
	Shaping knowledge	✓	✓	✓	✓	—	✓	✓	—	✓	✓	✓
	Regulations	✓	✓	—	—	—	—	—	—	—	✓	—

^a^Study ID in Multimedia Appendix 5.

^b^WM: weight loss maintenance interventions.

^c^WM7: the intervention [54] was also based on motivational theories, aspects of human motivation, and behavior change, not explicitly described.

^d^No behavior change technique (BCT) or theory was mentioned applied.

^e^BCT or theory applied.

^f^A: BCT or theory mentioned applied to stimulate adherence.

^g^M: BCT or theory mentioned applied to stimulate motivation.

**Figure 3:**
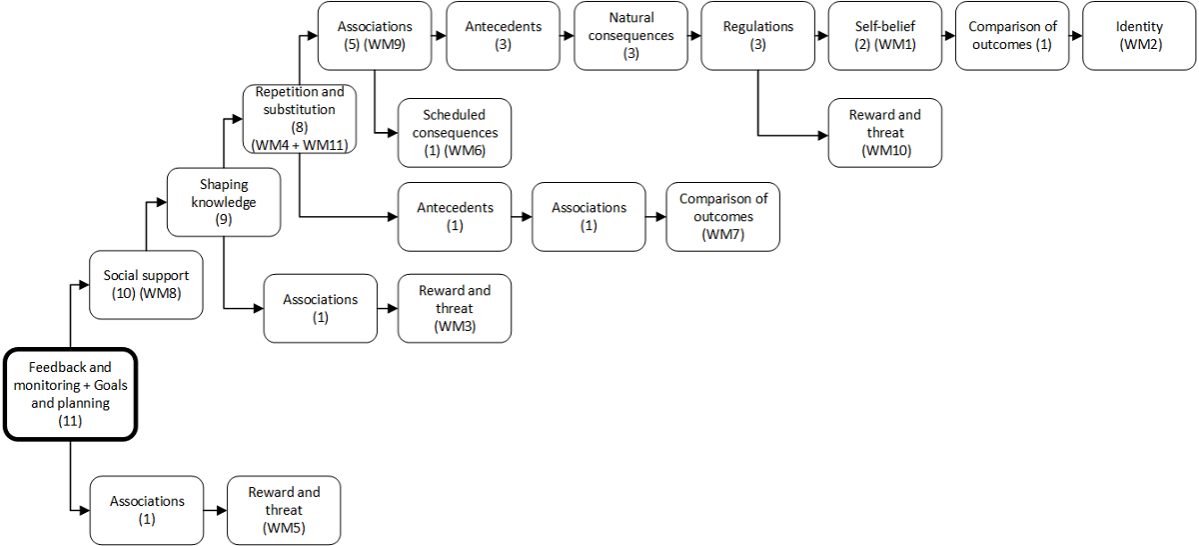
Flowchart with combinations of behavior change techniques (BCTs) in weight loss maintenance (WM) interventions 1-11. Illustrates the number (n) of WM interventions applying the BCT combined with previous techniques to the left in the flowchart.

Despite linking individual BCTs to stimulation of adherence and/or motivation, as indicated in Table 5, the weight loss maintenance interventions included in the review did not specify the ideal combination of such techniques. The most frequently applied combinations of BCTs in the 11 weight loss maintenance interventions are illustrated in Figure 3. This figure illustrates, from the *bold square* left to right, how many maintenance interventions (n) that actually applied the BCTs, in combination with the previous ones. The combinations *goals and planning* and *feedback and monitoring* were applied in all interventions and were, as indicated in the flowchart (Figure 3), frequently combined with *social support* (91%), *shaping knowledge* (82%), and *repetition and substitution* (73%). A handful of publications described *goals and planning* and *feedback and monitoring* as key strategies or core self-regulation techniques for behavior change and weight outcome and therefore applied these clusters [51,52,54,74,77]. A few publications described *social support* as important for motivation and engagement, reflected in the application of behavioral strategies and the intervention content [51,53]. *Social support*, provided by professionals or peers, was enabled in various ways with or without technology or in combination (blended care) [50,52-54,56,59]. *Shaping knowledge* was present in several ways through offering general suggestions and theory-based advice related to weight loss maintenance [50,51,54,56,58,59]. Finally, the use of *repetition and substitution* typically included habit formation, graded tasks, and behavioral rehearsal [47,51,57,59]. When identifying a specific weight loss maintenance intervention in Figure 3, WM1 to WM11, the exact BCTs applied in the weight loss maintenance intervention can be identified by following the reverse flow to the bold square to the left.

### Persuasive System Design Principles Used to Stimulate Motivation and Adherence in Electronic Health Weight Loss Maintenance Interventions

#### Persuasive System Design Principles and Motivation

The most frequently mentioned PSD principles applied to stimulate motivation in the weight loss maintenance interventions were, as indicated in Table 6, *personalization* (45%), *praise* (45%), and *feedback* (36%). Although *self-monitoring* was used in all included weight loss maintenance interventions and *goal setting* was used in 90% of the interventions, these principles were only mentioned applied once for the purpose to stimulate motivation [53,58].

The weight loss maintenance intervention where the most PSD principles were identified was an advanced gamified smartphone app where characters go through difficult situations, learning to cope with tempting situations (eg, social settings and holidays), and receive *rewards* through healthy habit points [47]. This intervention mentioned stimulating motivation through *tailoring*, *personalization*, and *praise*, for example, motivational messages or cognitive behavioral coping strategies depending on the challenges or situation [47].

Several technologies supporting weight loss maintenance stimulated motivation through motivational *feedback* messages and *praise*, often *personalized* and automated [47,51,53,54,56,57,62]. These messages were applied to motivate the user to stay on course or to provide support on good or bad days, often connected to self-reported feelings, weight (eg, when weight enters a *red zone*), or behaviors related to activities or food. In addition, 1 intervention delivered *tailored*, motivational messages, and coping *suggestions* through gaming elements to learn and *simulate* healthy behaviors [47].

#### Persuasive System Design Principles and Adherence

*Feedback* and *rewards* were persuasive principles mentioned to stimulate adherence (eg, 1 intervention used financial *rewards* to stimulate adherence to weekly weight loss maintenance goals) [55]. *Reminders* (27%) were often applied as automated notifications to submit *self-monitoring* information [47,52,57,59] or *remind* users about goals [51,53], although not explicitly mentioned as being applied to stimulate adherence [54,56,57]. *Feedback* and *reminders* (eg, when system usage decreased or when entering of monitoring data was required) and *tailoring* and *personalization* (eg, goal setting and system preferences) were used to meet the individual needs to stimulate adherence [53,54,56,57].

#### Persuasive System Design Principles Applied

Several PSD principles were applied in the weight loss maintenance interventions, as presented in Table 6, although usually not explicitly mentioned applied with the purpose of stimulating motivation and adherence in particular.

**Table 6:** Included weight loss maintenance interventions specifying persuasive system design (PSD) principles.

Study ID		WM^a^ 1 [57]	WM 2 [51]	WM 3 [52]	WM 4 [59]	WM 5 [55]	WM 6 [56]	WM 7 [54]	WM 8 [53]	WM 9 [50]	WM 10 [47]	WM 11 [58]
**Primary task support**												
	Reduction	—^b^	—	—	—	—	✓^c^	✓	—	—	✓	—
	Tunneling	—	—	—	—	—	✓	✓	—	—	✓	—
	Tailoring	✓	✓	✓	✓	✓	✓	✓	M^d^ / A^e^	✓	M	✓
	Personalization	✓	M	—	—	—	A	M	M	✓	M	✓
	Self-monitoring	✓	✓	✓	✓	✓	✓	✓	✓	✓	✓	M
	Simulation	✓	✓	—	✓	—	✓	✓	✓	✓	✓	—
	Rehearsal	—	—	—	—	—	✓	—	—	—	✓	—
**Dialog support**												
	Praise	M	M	—	—	—	—	—	M	—	M	—
	Rewards	—	—	A	✓	M / A	✓	✓	—	—	✓	—
	Reminders	✓	✓	✓	✓	✓	✓	✓	✓	—	✓	—
	Suggestions	✓	—	—	✓	—	✓	✓	✓	—	✓	✓
	Similarity	—	—	—	—	—	—	—	—	—	—	—
	Liking	—	—	—	—	—	—	—	—	—	—	—
	Social role	—	—	—	—	—	—	—	—	—	✓	—
**Social support**												
	Social learning	—	—	—	—	—	—	✓	—	—	—	—
	Social comparison	—	—	—	—	—	—	✓	—	—	✓	—
	Normative influence	—	—	—	—	—	—	—	—	—	—	—
	Social facilitation	—	—	✓	—	—	—	✓	—	—	—	—
	Cooperation	—	—	—	—	—	—	✓	—	—	—	—
	Competition	—	—	—	—	—	—	—	—	—	—	—
	Recognition	—	—	—	—	—	—	—	—	—	✓	—
**Other**												
	Feedback	M / A	M	✓	✓	✓	A	✓	M	✓	M	✓
	Goal setting	✓	✓	✓	✓	✓	✓	✓	M	✓	—	✓
	Social support	—	✓	✓	—	—	✓	✓	M / A	✓	—	✓

^a^WM: weight loss maintenance intervention.

^b^No PSD was identified applied.

^c^PSD identified.

^d^M: PSD mentioned applied to stimulate motivation.

^e^A: PSD mentioned applied to stimulate adherence.

#### Operationalization of Commonly Applied Persuasive System Design Principles

Reasons to apply the *self-monitoring* principle were because users could monitor weight, diet, and/or activity, often related to short- or long-term goals [51,53,57], or *self-monitoring* of mood, stress, and/or habit tracking [47,51,53,57]. Monitoring data were registered automatically as well as manually (eg, through wireless trackers and scales) or using the website to enter weight, activity, and diet data [50,54,55]. *Goal setting* was often integrated into the technology, allowing users to set, monitor, or review both short- and long-term goals (sometimes through *reduction*), related to the behavior they wanted to change [50-56,58,59]. *Tailoring* of messages or *feedback* to the users were again often linked to *self-monitoring* of weight, diet, and activity information, as participants received *tailored feedback* on their progress [51,52,56].

#### Persuasive System Design Combinations

**Figure 4:**
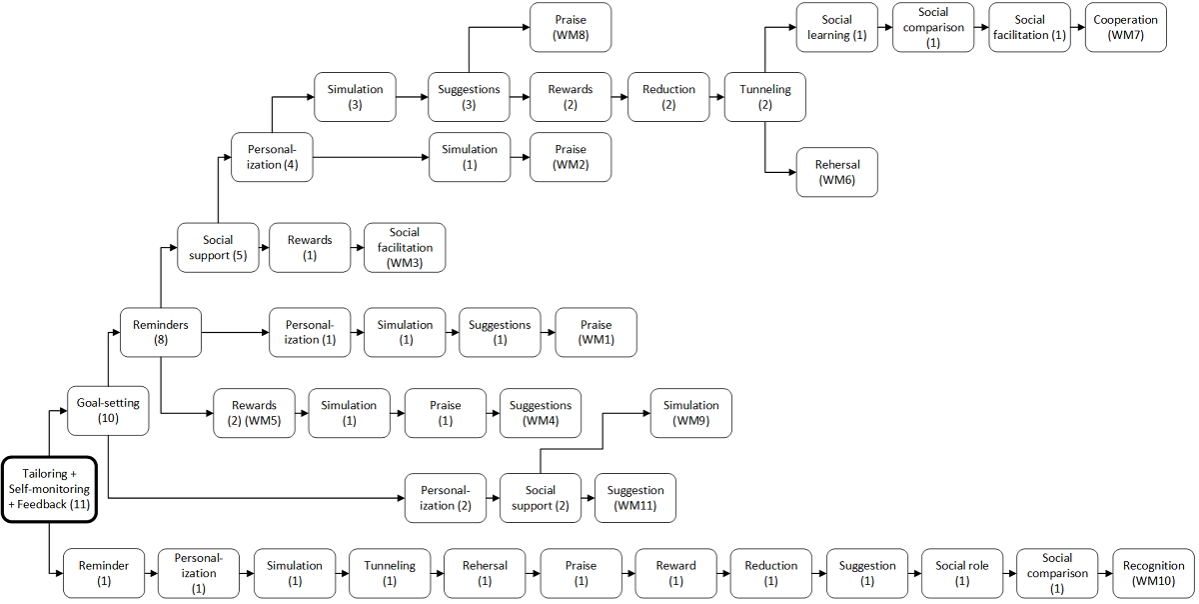
Flowchart with combinations of persuasive system design (PSD) principles used in weight loss maintenance (WM) interventions 1-11. Illustrates the number (n) of WM interventions applying the PSD principles combined with previous principles to the left in the flowchart.

The most frequent combinations of PSD principles used in all 11 weight loss maintenance interventions were *tailoring*, *self-monitoring,* and *feedback* (100%). As the flowchart in Figure 4 indicates, 90% of the interventions combined these frequently used features with *goal setting* (90%) and *reminders* (73%). The flowchart provides an overview of the number of maintenance interventions (n) that actually applied the various PSD principles, in combination with the previous ones.

### Weight Loss Maintenance Effects (Weight Outcomes)

Of the 11 included interventions targeting weight loss maintenance, only 3 evaluated effects of the intervention on body weight or BMI. All 3 interventions found significant effects for weight loss maintenance at 12 weeks postbaseline [59], 12 months postintervention [52], and up to 24 months postbaseline [50].

In addition, one of the interventions had a 6-month weight loss phase followed by a 6-month weight loss maintenance phase and presented characteristics of *high-performing* participants who had lost 10% or greater of their starting weight at the 1-year follow-up [50]. High performers compared with low performers had greater adherence to *self-monitoring* of weight, more days wearing activity trackers, and higher average number of steps per day [50]. In another study, *rewards* were applied to stimulate adherence, suggesting that an internet-delivered cost-benefit approach might be effective to support weight loss maintenance [52]. The third study, entailing a mobile health (ie, mobile or cellular phone technology) lifestyle program, implemented weekly text messages to prevent weight gain, using *praise* to stimulate motivation [59]. This intervention appeared successful in preventing unhealthy weight gain, resulting in modest weight loss and improved health behaviors [59].

The 3 weight loss maintenance interventions achieving effect in terms of weight outcomes were similar in that they all applied techniques and principles related to *tailoring*, *self-monitoring*, *feedback*, *goals and planning*, and *shaping knowledge*. A human *social support* component delivered through a blended format by e-coaching [52], telephone support [59], or expert coach by a Web-based electronic messaging feature [50] was also present in all 3 interventions.

## Discussion

### Principal Findings

This scoping review aimed to identify BCTs and PSD principles applied in eHealth interventions to support weight loss and weight loss maintenance, as well as techniques and principles applied to stimulate *motivation* and *adherence* for long-term weight loss maintenance. The most successful eHealth weight loss maintenance interventions entailed a combination of BCTs and PSD principles, and the analysis identified several techniques and principles applied to stimulate *motivation* and *adherence*.

#### Adherence and Motivation

Focus on and description of *motivation* and *adherence* were more prominent in the included weight loss maintenance interventions than in the weight loss interventions. Only 2 of the 45 publications described a definition for *motivation,* and *motivation* was measured in the weight loss interventions but not in the weight loss maintenance interventions. The results provided some indications that the delivery of tailored, real-time daily feedback messages related to diary entries could enhance motivation [67], use of a virtual coach could be used to motivate users to become more active [49], and autonomous motivation was predictive for adherence to self-monitoring [44]. *Adherence* was defined and measured in various ways in both types of interventions, including but not limited to behavioral adherence, program compliance, technology usage, or adherence to certain technology features (eg, self-monitoring). The evaluation methods applied to measure adherence did not focus on evaluating actual use of the technology but only usage of certain technology features (eg, self-monitoring). The results indicated that *adherence* or usage of self-monitoring techniques was associated with weight loss [50,68,69,74]. The findings related to both *motivation* and *adherence* may provide an interesting input for eHealth development but makes it challenging to compare results across interventions because of diversity in study designs and reporting.

#### Behavior Change Theories, Behavior Change Techniques, and Persuasive System Design Principles Applied in Weight Loss and Weight Loss Maintenance Interventions

The most frequently used technology in the included interventions of this review was mobile phone, often used for monitoring, dialog, feedback, and support. All interventions had a theoretical anchoring and applied various BCTs and PSD principles. The analysis revealed that techniques and principles applied to support behavior change in weight loss interventions do not necessarily equal weight loss maintenance. However, some key BCTs and PSD principles, identified by applying the Michie’s Behavior Change Taxonomy and the PSD model [14,82], including *goal setting*, *self-monitoring*, *feedback*, and *shaping knowledge*, were present in most of the included interventions. The PSD principles from the *primary task support* and *dialog support category* were most frequently applied. *Social support* was also identified as a frequently applied BCT in both types of interventions. Within the PSD model, *social support* was set as a separate PSD principle, as it was difficult to identify within the *social support category* based on the information provided in the publications.

##### Weight Loss Interventions

The typical weight loss interventions were usually of shorter duration than the weight loss maintenance interventions. *Social cognitive theory* was the most commonly applied behavior change theory in weight loss interventions. The identified core techniques and principles mentioned were used in the technology to support target behavior (weight loss). BCTs and PSD principles more frequently applied in weight loss than weight loss maintenance interventions included *comparison of behavior* and *competition* often to motivate or inspire healthy attitudes and performance between users of the technology (eg, sharing progress, weight changes, and targets achieved).

##### Weight Loss Maintenance Interventions

The identified 11 weight loss maintenance interventions included in this review had a duration range between 12 and 52 weeks, were often presented as a protocol or described the design and development process only. Of 11 weight loss maintenance interventions, only 3 focused on evaluating weight loss maintenance effects. *Self-regulation theory* was the most often mentioned applied behavior change theory in weight loss maintenance interventions. The core BCTs and PSD principles (eg, *self-monitoring*, *feedback*, *goals and planning*, *tailoring,* and *shaping knowledge*) were reflected in the technology design and considered important for behavior change and weight loss maintenance. Although the ideal combination of BCTs or PSD principles to stimulate motivation, adherence and weight loss maintenance was not explicitly stated, the most frequently mentioned techniques and persuasive principles applied to stimulate motivation were *personalization*, *praise*, and *feedback*, whereas *associations* were frequently mentioned to stimulate adherence. *Rewards* and *social support* were used to stimulate both motivation and adherence. Technologies applying techniques and principles supporting behaviors to deal with biological, environmental, social, behavioral, and cognitive factors (eg, creation of self-determined goals related to healthy habits and self-monitoring) were represented in many of the included weight loss maintenance interventions. In the maintenance phase, s*ocial support*, *rewards*, *reduction*, *praise*, *repetition and substitution,* and *prompts and cues* could be of particular importance in addition to the core techniques identified to address the cost-benefit ratio by incentive driven, rewarding, and persuasive technologies.

The findings in this review are in line with earlier research indicating that behavioral strategies may facilitate health behavior change to maintain weight loss [4,86-88] but that more research focusing on long-term eHealth weight loss maintenance is needed [27,35,89-92].

A recent systematic review on determinants of weight loss maintenance confirmed that evidence related to motivation is sparse [93], and further evidence is needed. Standardization of the adherence concept and reporting [94,95] may also contribute to open the *black box* of eHealth to understand how design and use of eHealth technologies may contribute to improved health and well-being [30].

Lack of information on the *social support* and the *system credibility support* categories have also been pointed to as sparsely reported on by an earlier review on key components in eHealth interventions promoting healthier lifestyle [96]. Earlier research has shown that these categories are important to include when reporting, as users have been less engaged with the technology if credibility was lacking, which again can affect health behavior [97]. This identifies a need for more diligent reporting on design of eHealth interventions and a need for investigation as to which design elements are actually required to achieve behavior change are needed.

This review also shows a lack of user involvement in several of the included interventions. To develop effective eHealth interventions, orchestrated content and system development are needed, as these are often separated by a variety of strategies initiated by researchers and designers of technologies. These challenges can be overcome by multidisciplinary and interwoven human-centered design approaches during the development of eHealth technologies aimed to change behaviors [12,25].

Although evidence related to theoretical explanation of sustainable maintenance of behavior change is limited [88], existing reviews of technologies point to the need for combinations of BCTs and PSDs to achieve successful health behavior change and weight management [35,91,96,98]. Existing research also points to frequent *self-monitoring* of weight and food intake, high levels of physical activity [87,99,100], and healthy diet as key ingredients often present in weight loss maintenance interventions associated with better weight loss maintenance over time [4,5,8,93,100-103].

Digital developments bring several design opportunities that allow for development and testing of meaningful, adaptive, and sustainable health-promoting solutions [25]. Integration of persuasive interaction and design elements (eg, gaming, avatars, and virtual coach) to reward, rehearse, or simulate cognitive, social, and biological aspects of healthy behaviors or attitudes can provide new methods to learn and maintain new lifestyle and the lost weight, as establishment of healthy behaviors takes time [102]. As smart monitoring is evolving and automatic tracking devices are available in almost all smartphones, this can allow for personalized feedback and long-term monitoring of wellness goals related to a healthy lifestyle that can be maintained lifelong.

### Recommendation for Future Design and Research

First, research into design and application of new, personalized digital technologies that integrate sensors and long-term monitoring of data of behaviors and decisions can provide opportunities that may contribute to ultimately solve the conundrum of sustainable health behavior change and long-term weight loss maintenance. Second, the identification of central BCTs and PSD principles to support behavior change, motivation, and adherence in this review allow for user testing in predesign phases of behavioral eHealth interventions, which again can aid in the evaluation of what is needed to truly support individuals in their health. This review also identified self-regulation techniques to support creation and maintenance of healthy habits, but the ideal combination of such techniques should be further investigated through design and evaluation of novel technologies to support long-term weight maintenance after weight loss. Building healthy habits and behaviors takes time, and future research should explore how personalized eHealth technologies can support patients’ motivation, long-term adherence, and sustained engagement to improve healthy behaviors over time.

Future research can also better facilitate comparison of interventions through following standardized guidelines and frameworks more diligently when reporting findings, including following guidelines and frameworks such as the CONSORT Guidelines [39], the BCT Taxonomy of Michie [21], and/or the PSD model by Oinas-Kukkonen [16].

Finally, eHealth interventions developed in line with user values and needs may have the potential to motivate and empower sustainable health behavior change, which calls for more user involvement and multidisciplinary approaches in design, development, and evaluation of eHealth interventions. Such an interwoven development process, combining the input, needs, and requirements of researchers, engineers, and stakeholders (including users), is needed to unravel or disentangle the *black box* and create technologies that engage, motivate, and support health behaviors that can be sustained to maintain lost weight for a lifetime.

### Strengths and Limitations

This review has a number of limitations. First, identification and scoring of the persuasive features and BCTs could have been prone to subjectivity by the researcher(s). However, to prevent subjectivity, consultation and a 10% validation of included publications were performed. Second, *system credibility support* was not included in the analyses, as limited data were reported and examinations of the actual technology were not included in this review. However, the analysis reveals knowledge about the other 3 PSD categories, in particular, the *primary tasks* and *dialog support*. Third, no quality appraisal of evidence, often done in systematic reviews, was performed in this scoping review. This limits the possibility of drawing conclusions regarding cause and (long-term) effectiveness of interventions. However, as the aim of this scoping review was to provide insight into the design of eHealth interventions, particularly the PSD principles and BCTs mentioned applied to support sustained behavior change and weight loss maintenance, a quality assessment of the studies included was not considered to be as relevant. In addition, insight into various study designs provides an overview over this emerging research area. Finally, the heterogeneity of included designs and lack of long-term results complicate a comparison of the interventions and the possible impact of techniques and principles on reported outcomes, which may introduce bias.

The focus on weight loss maintenance is, however, a major strength of this scoping review, as weight loss maintenance is an area in dire need of further research and future recommendations. In addition, the inclusion of a variety of study designs allows for a consideration of existing interventions that describe design choices and formative evaluations, contributing to eventually opening up the *black box* and giving direction for future design of eHealth interventions.

### Conclusions

To the best of our knowledge, this review is the first to identify BCTs and PSD principles applied in eHealth weight loss and weight loss maintenance interventions. Results reveal very limited existing research in the area of eHealth interventions to support weight loss maintenance. *Motivation* and *adherence* are clearly of essence in terms of achieving long-term weight loss maintenance, yet there is still a lack of standardization in definitions and measurement of these concepts. Results show how self-regulation strategies are applied in weight loss and weight loss maintenance interventions, reflected in the design through core techniques and principles such as *self-monitoring*, *feedback*, *goals and planning*, *tailoring*, and *shaping knowledge*. Frequently mentioned BCTs and PSD principles applied to stimulate *motivation* in weight loss maintenance interventions were *personalization*, *praise*, and *feedback*, whereas *associations* were mentioned to stimulate adherence. *Social support* and *rewards* were mentioned as being applied to stimulate both motivation and adherence. The most effective combination of techniques or design features to stimulate *motivation*, *adherence*, and weight loss maintenance nevertheless remains somewhat obscure. Although few weight loss maintenance eHealth interventions indicated effect (ie, weight outcome), the interventions with significant results all applied the identified core BCTs and PSD principles, as well as a human *social support* component.

In conclusion, this scoping review aimed to contribute to open the *black box* of eHealth in the design of weight loss maintenance interventions. The findings are expected to contribute to a better understanding of existing research in this field, and in addition to contribute to development and evaluation of future eHealth interventions and novel solutions to support sustained behavior change and long-term weight loss maintenance. The results of this review support the notion that the research of eHealth interventions in weight loss maintenance is still in its infancy, and more research is needed.

## Appendix

Multimedia Appendix 1 Cluster label and component behavior change techniques (Michie et al, 2013).

Multimedia Appendix 2 The persuasive system design model (Oinas-Kukkonen, 2009).

Multimedia Appendix 3 Search strategy.

Multimedia Appendix 4 Inclusion and exclusion criteria.

Multimedia Appendix 5 Overview of included publications.

Multimedia Appendix 6 Reported outcomes.

Multimedia Appendix 7 Overview over behavior change theories and techniques used in the included eHealth interventions.

Multimedia Appendix 8 Overview over persuasive system design principles used in the included eHealth interventions.

## Abbreviations

BCT: behavior change techniques
BMI: body mass index
eHealth: electronic health
PSD: persuasive system design
