# Supplementary material for: Persuasive System Design Principles and Behavior Change Techniques to Stimulate Motivation and Adherence in Electronic Health Interventions to Support Weight Loss Maintenance: Scoping Review
Source: J Med Internet Res. 2019 Jun 21;21(6):e14265. doi: 10.2196/14265 (PMC6611151; doi:10.2196/14265)
Supplement: Multimedia Appendix 1 [file jmir_v21i6e14265_app1.pdf]

Multimedia Appendix 1 Cluster label and component BCTs AU, % (SE) (Michie et al, 2013)

(1) Scheduled consequences 91 (.004)
- Punishment [14.2]
- Response cost [14.1]
- Chaining [14.5]
- Extinction [14.3]
- Discrimination training [14.6]
- Shaping [14.4]
- Negative reinforcement [14.10]
- Counter-conditioning [14.7]
- Thinning [14.9]
- Differential reinforcement [14.8]

(**2) Reward and threat** 90 (.005)
- Social reward [10.4]
- Material reward [10.2]
- Self-reward [10.9]
- Non-specific reward [10.3]
- Threat [10.11]
- Anticipation of future rewards or removal of punishment [14.10]
- Incentive [10.1]

(3) Repetition and substitution 97 (.002)
- Behavior substitution [8.2]
- Habit reversal [8.4]
- Habit formation [8.3]
- Graded tasks [8.7]
- Overcorrection [8.5]
- Behavioral rehearsal/practice [8.1]
- Generalization of a target behavior [8.6]

(4) Antecedents 96 (.002)
- Restructuring the physical environment [12.1]
- Restructuring the social environment [12.2]
- Avoidance/changing exposure to cues for the behavior
[12.3]
- Distraction [12.4]

(5) Associations 97 (.002)
- Discriminative (learned) cue [7.2]
- Time out [7.4]
- Escape learning [7.5]
- Satiation [7.6]
- Exposure [7.7]
- Classical conditioning [7.8]
- Fading [7.3]
- Prompts/cues [7.1]

(6) Covert learning 73 (.008)
- Vicarious reinforcement [16.3]
- Covert sensitization [16.1]
- Covert conditioning [16.2]

(7) Natural consequences 85 (.006)
- Cluster label and component BCTs AU, % (SE)
- Health consequences [5.1]
- Social and environmental consequences [5.3]
- Salience of consequences [5.2]
- Emotional consequences [5.6]
- Self-assessment of affective consequences [5.4]
- Anticipated regret [5.5]

(8) Feedback and monitoring 97 (.002)
- Feedback on behavior [2.2]
- Biofeedback [2.6]
- Other(s) monitoring with awareness [2.1 and 2.5]
- Self-monitoring of outcome of behavior [2.4]
- Self-monitoring of behavior [2.3]

(9) Goals and planning 90 (.002)
- Action planning (including implementation intentions)
[1.4]
- Problem solving/coping planning [1.2]
- Commitment [1.9]
- Goal setting (outcome) [1.3]
- Behavioral contract [1.8]
- Discrepancy between current behavior and goal
standard [1.6]
- Goal setting (behavior) [1.1]
- Review behavior goal(s) [1.5]
- Review of outcome goal(s) [1.7]

(10) Social support 100 (.001)
- Social support (practical) [3.2]
- Social support (general) [3.1]
- Social support (emotional) [3.3]

(11) Comparison of behavior 83 (.006)
- Modeling of the behavior [6.1]
- Information about others' approval [6.3]
- Social comparison [6.2]

(12) Self-belief 92 (.005)
- Mental rehearsal of successful performance [15.2]
- Self-talk [15.4]
- Focus on past success [15.3]
- Verbal persuasion to boost self-efficacy [15.1]

(13) Comparison of outcomes 90 (.005)
- Persuasive argument [9.1]
- Pros and cons [9.2]
- Comparative imagining of future outcomes [9.3]

(14) Identity 86 (.006)
- Identification of self as role model [13.1]
- Self-affirmation [13.4]
- Identity associated with changed behavior [13.5]
- Reframing [13.2]
- Cognitive dissonance [13.3]

(15) Shaping knowledge 95 (.003)
- Reattribution [4.3]
- Antecedents [4.2]
- Behavioral experiments [4.4]
- Instruction on how to perform a behavior [4.1]

(16) Regulation 98 (.001)
- Regulate negative emotions [11.2]
- Conserving mental resources [11.3]
- Pharmacological support [11.1]
- Paradoxical instructions [11.4]
