# Supplementary material for: Persuasive System Design Principles and Behavior Change Techniques to Stimulate Motivation and Adherence in Electronic Health Interventions to Support Weight Loss Maintenance: Scoping Review
Source: J Med Internet Res. 2019 Jun 21;21(6):e14265. doi: 10.2196/14265 (PMC6611151; doi:10.2196/14265)
Supplement: Multimedia Appendix 2 [file jmir_v21i6e14265_app2.pdf]

Multimedia Appendix 2: The PSD model (Oinas-Kukkonen, 2009)

Primary Task Support		
**Principle**	**Example requirement**	**Example implementation**
**Reduction**<br>A system that reduces complex behavior into simple tasks helps users perform the target behavior, and it may increase the benefit/cost ratio of a behavior.	System should reduce effort that users expend with regard to performing their target behavior.	Mobile application for healthier eating habits lists proper food choices at fast food restaurants [Lee et al. 2006]. Smoking cessation Web site provides an interactive test that measures how much money a user will save with quitting.
**Tunneling**<br>Using the system to guide users through a process or experience provides opportunities to persuade along the way.	System should guide users in the attitude change process by providing means for action that brings them closer to the target behavior.	Smoking cessation Web site offers information about treatment opportunities after a user has taken an interactive test about how addicted (s)he is on tobacco.
**Tailoring**<br>Information provided by the system will be more persuasive if it is tailored to the potential needs, interests, personality, usage context, or other factors relevant to a user group.	System should provide tailored information for its user groups.	Personal trainer Web site provides different information content for different user groups, e.g. beginners and professionals. Web site for recovering alcoholics presents stories that are close to the user's own story.
**Personalization**<br>A system that offers personalized content or services has a greater capability for persuasion.	System should offer personalized content and services for its users.	Arguments most likely to be relevant for the user presented first on a professional Web site rather than in random order.
**Self-monitoring**<br>A system that keeps track of one's own performance or status supports the user in achieving goals.	System should provide means for users to track their performance or status.	Heart rate monitor presents a user's heart rate and the duration of the exercise. Mobile phone application presents daily step count [Consolvo et al. 2006].
**Simulation**<br>Systems that provide simulations can persuade by enabling users to observe immediately the link between cause and effect.	System should provide means for observing the link between the cause and effect with regard to users' behavior.	Before-and-after pictures of people who have lost weight are presented on a Web site.
**Rehearsal**<br>A system providing means with which to rehearse a behavior can enable people to change their attitudes or behavior in the real world.	System should provide means for rehearsing a target behavior.	A flying simulator to help flight pilots practice for severe weather conditions.

Dialogue Support		
**Principle**	**Example requirement**	**Example implementation**
**Praise**<br>By offering praise, a system can make users more open to persuasion.	System should use praise via words, images, symbols, or sounds as a way to provide user feedback information based on his/her behaviors.	Mobile application that aims at motivating teenagers to exercise praises user by sending automated text messages for reaching individual goals. [Toscos et al. 2006]
**Rewards**<br>Systems that reward target behaviors may have great persuasive powers.	System should provide virtual rewards for users in order to give credit for performing the target behavior.	Heart rate monitor gives users a virtual trophy if they follow their fitness program. Game rewards users by altering media items, such as sounds, background skin, or a user's avatar according to user's performance. [Sohn and Lee 2007]
**Reminders**<br>If a system reminds users of their target behavior, the users will more likely achieve their goals.	System should remind users of their target behavior during the use of the system.	Caloric balance monitoring application sends text messages to its users as daily reminders. [Lee et al.2006]
**Suggestion**<br>Systems offering fitting suggestions will have greater persuasive powers.	System should suggest that users carry out behaviors during the system use process.	Application for healthier eating habits suggests that children eat fruits instead of candy at snack time.
**Similarity**<br>People are more readily persuaded through systems that remind them of themselves in some meaningful way.	System should imitate its users in some specific way.	Slang names are used in an application which aims at motivating teenagers to exercise. [Toscos et al. 2006]
**Liking**<br>A system that is visually attractive for its users is likely to be more persuasive.	System should have a look and feel that appeals to its users.	Web site that aims at encouraging children to take care of their pets properly has pictures of cute animals.
**Social role**<br>If a system adopts a social role, users will more likely use it for persuasive purposes.	System should adopt a social role.	E-health application has a virtual specialist to support communication between users and health specialists. [Silva et al. 2006]

System Credibility Support		
**Principle**	**Example requirement**	**Example implementation**
**Trustworthiness**<br>A system that is viewed as trustworthy will have increased powers of persuasion.	System should provide information that is truthful, fair and unbiased.	Company Web site provides information related to its products rather than simply providing biased advertising or marketing information.
**Expertise**<br>A system that is viewed as incorporating expertise will have increased powers of persuasion.	System should provide information showing knowledge, experience, and competence.	Company Web site provides information about their core knowledge base. Mobile application is updated regularly and there are no dangling links or out-of-date information.
**Surface credibility**<br>People make initial assessments of the system credibility based on a firsthand inspection.	System should have competent look and feel.	There are only a limited number of, and a logical reason for, ads on a Web site or mobile application.
**Real-world feel**<br>A system that highlights people or organization behind its content or services will have more credibility.	System should provide information of the organization and/or actual people behind its content and services.	Company Web site provides possibilities to contact specific people through sending feedback or asking questions.
**Authority**<br>A system that leverages roles of authority will have enhanced powers of persuasion.	System should refer to people in the role of authority.	Web site quotes an authority, such as a statement by government health office.
**Third-party endorsements**<br>Third-party endorsements, especially from well-known and respected sources, boost perceptions on system credibility.	System should provide endorsements from respected sources.	E-shop shows a logo of a certificate that assures that they use secure connections. Web site refers to its reward for high usability.
**Verifiability**<br>Credibility perceptions will be enhanced if a system makes it easy to verify the accuracy of site content via outside sources.	System should provide means to verify the accuracy of site content via outside sources.	Claims on a Web site are supported by offering links to other web sites.

Social Support		
**Principle**	**Example requirement**	**Example implementation**
**Social learning**<br>A person will be more motivated to perform a target behavior if (s)he can use a system to observe others performing the behavior.	System should provide means to observe other users who are performing their target behaviors and to see the outcomes of their behavior.	A shared fitness journal in a mobile application for encouraging physical activity [Consolvo et al. 2006].
**Social comparison**<br>System users will have a greater motivation to perform the target behavior if they can compare their performance with the performance of others.	System should provide means for comparing performance with the performance of other users.	Users can share and compare information related to their physical health and smoking behavior via instant messaging application [Sohn and Lee 2007].
**Normative influence**<br>A system can leverage normative influence or peer pressure to increase the likelihood that a person will adopt a target behavior.	System should provide means for gathering together people who have the same goal and make them feel norms.	A smoking cessation application shows pictures of newborn babies with serious health problems due to the mother's smoking habit.
**Social facilitation**<br>System users are more likely to perform target behavior if they discern via the system that others are performing the behavior along with them.	System should provide means for discerning other users who are performing the behavior.	Users of a computer-based learning environment can recognize how many costudents are doing their assigned homework at the same time as them.
**Cooperation**<br>A system can motivate users to adopt a target attitude or behavior by leveraging human beings' natural drive to co-operate.	System should provide means for co-operation.	The behavioral patterns of overweight patients are studied through a mobile application, which collects data and sends it to a central server where it can be analyzed at the group level in more detail [Lee et al. 2006].
**Competition**<br>A system can motivate users to adopt a target attitude or behavior by leveraging human beings' natural drive to compete.	System should provide means for competing with other users.	Online competition, such as Quit and Win (stop smoking for a month and win a prize).
**Recognition**<br>By offering public recognition for an individual or group, a system can increase the likelihood that a person/ group will adopt a target behavior.	System should provide public recognition for users who perform their target behavior.	Names of awarded people, such as "stopper of the month," are published on a Web site. Personal stories of the people who have succeeded in their goal behavior are published on a smoking cessation Web site.
