# Supplementary material for: Persuasive System Design Principles and Behavior Change Techniques to Stimulate Motivation and Adherence in Electronic Health Interventions to Support Weight Loss Maintenance: Scoping Review
Source: J Med Internet Res. 2019 Jun 21;21(6):e14265. doi: 10.2196/14265 (PMC6611151; doi:10.2196/14265)
Supplement: Multimedia Appendix 3 [file jmir_v21i6e14265_app3.pdf]

Multimedia Appendix 3 Search strategy

#	Search strategy:
1	(obes* or overweight or (weight adj1 (maintenance or stabilisation or stabilization or management or loss))).tw.
2	(e?health or e health or m?health or m health or online or mobile health or telemedicine or social media or web based or smartphone* or ((mobile or cellular or smart) adj phone*) or ((mobile or cellular or internet or smartphone*) adj3 (app* or technology or intervention))).tw.
3	(motivat* or adhere* or compliance).tw.
4	(behavio* adj1 chang*).tw.
5	1 and 2 and 3 and 4
6	remove duplicates from 5
7	limit 6 to yr="2007 -Current"
