# Supplementary material for: Persuasive System Design Principles and Behavior Change Techniques to Stimulate Motivation and Adherence in Electronic Health Interventions to Support Weight Loss Maintenance: Scoping Review
Source: J Med Internet Res. 2019 Jun 21;21(6):e14265. doi: 10.2196/14265 (PMC6611151; doi:10.2196/14265)
Supplement: Multimedia Appendix 4 [file jmir_v21i6e14265_app4.pdf]

Multimedia Appendix 4 Inclusion and exclusion criteria

<u>Inclusion criteria</u>

All five criteria (1, 2, 3, 4 and 5) must be met for a research article to be included, together with criteria 6 OR 7:

1. Goal of the technology intervention: Weight maintenance OR weight loss
2. Population: Target group for the intervention are patients struggling with overweight or obesity and informal caregivers
3. The patient must be able to self-manage the technology / eHealth / web-based intervention / mHealth / telemedicine
4. Participants / users: >18 years' old (the technology was not specifically designed for adolescents or children under 18 years)
5. The study is peer-reviewed and published
6. The (primary) study mention: adherence / compliance / motivation to the technology / eHealth / web-based intervention
7. The article describes a (protocol for a) primary study where persuasive design OR persuasive design features OR behavior change theories OR behavior change models / techniques are mentioned

<u>Exclusion criteria</u>

1. Goal of the technology interventions is not related to weight maintenance / weight loss as an outcome
2. Adherence to a study protocol or a survey or medication
3. Non-eHealth (or technology / intervention mentioned in inclusion criteria 3)
4. Other patient groups / population than overweight or obese patients
5. Participants / users: < 18 years´ old
6. The article is a review or conference abstract or full-text was not available
7. The (primary) study does not mention adherence / compliance / motivation to a technology / eHealth / web-based intervention
8. The article does not describe a (protocol for a) primary study where persuasive design OR persuasive design features OR behavior change theories OR behavior change models / techniques are mentioned
