# Supplementary material for: Persuasive System Design Principles and Behavior Change Techniques to Stimulate Motivation and Adherence in Electronic Health Interventions to Support Weight Loss Maintenance: Scoping Review
Source: J Med Internet Res. 2019 Jun 21;21(6):e14265. doi: 10.2196/14265 (PMC6611151; doi:10.2196/14265)
Supplement: Multimedia Appendix 5 [file jmir_v21i6e14265_app5.pdf]

Multimedia Appendix 5 Overview included publications

☒ Outcome measured ☐ Outcome not measured

Intervention number	Outcomes measured	Interventions characteristics
**WM1**	Not applicable	**Title:** Combining Persuasive Technology With Behavioral Theory to Support Weight Maintenance Through a Mobile Phone App: Protocol for the MotiMate App<br>**Authors (year):** Brindal et al. (2016)<br>**Study design:** Protocol<br>**Objective:** To apply persuasive and behavioral theory in the development of an app targeting weight loss maintenance.<br>**Participants:** Volunteers (n=88)<br>**Country:** Australia<br>**Aim of technology:** Weight maintenance<br>**Type of technology or device:** Smartphone<br>**Intervention duration:** Not applicable<br>**Blended care:** No
**WM2**	Not applicable	**Title:** The NULevel trial of a scalable, technology-assisted weight loss maintenance intervention for obese adults after clinically significant weight loss: Study protocol for a randomized controlled trial<br>**Authors (year):** Evans et al. (2015)<br>**Study design:** RCT protocol<br>**Objective:** Protocol describe a RCT to evaluate the effectiveness and cost-effectiveness of a scalable, technology-assisted behavioral intervention for weight loss maintenance (WLM) in obese adults after initial weight loss<br>**Participants:** ≥18 years, BMI ≥ 30 with weight loss of ≥5% in the 12 months before study (n=288)<br>**Country:** United Kingdom<br>**Aim of technology:** Weight maintenance<br>**Type of technology or device:** Smartphone<br>**Intervention duration:** 12 months<br>**Blended care:** Yes (single face-to-face contact initially. On request individual telephone calls with a member of research team to discuss specific problems with WLM)
**WM3**	**Weight ☒**<br>**Adherence ☒**<br>**Motivation ☐**	**Title:** A randomized controlled trial testing an Internet delivered cost–benefit approach to weight loss maintenance<br>**Authors (year):** Leahey et al. (2016)<br>**Study design:** RCT<br>**Objective:** To examine the efficacy of a novel approach to weight loss maintenance based on modifying the cost-benefit ratio<br>**Participants:** 18–70 years, BMI>=25kg/m2, 5% weight loss threshold upfront (n=138)<br>**Country:** United States<br>**Aim of technology:** Weight maintenance<br>**Type of technology or device:** Web-based<br>**Intervention duration:** 10 months<br>**Blended care:** Yes (2 arms received one time group session, and a professional e-coach or a peer e-coach)

**WM4**	**Weight** ☒<br>**Adherence** ☒<br>**Motivation** ☐	**Title:** Effectiveness of a mHealth Lifestyle Program With Telephone Support (TXT2BFiT) to Prevent Unhealthy Weight Gain in Young Adults: Randomized Controlled Trial<br>**Authors (year):** Patridge et al. (2015)<br>**Study design:** RCT (2 arms)<br>**Objective:** Assesse the efficacy of TXT2BFIT program in preventing excess weight gain, improving dietary and physical activity behaviors in young adults at increased risk of obesity and unhealthy lifestyle choices.<br>**Participants:** 18-35 years, BMI 23-24,9 with 2 kg gain previous 12 months, or BMI 25-31,9 (n=250)<br>**Country:** Australia<br>**Aim of technology:** Weight maintenance<br>**Type of technology or device:** Smartphone<br>**Intervention duration:** 12 weeks<br>**Blended care:** Yes
**WM5**	Not applicable	**Title:** The design and conduct of Keep It Off: An online randomized trial of financial incentives for weight-loss maintenance<br>**Authors (year):** Shaw et al. (2017)<br>**Study design:** Protocol (RCT 3 arms)<br>**Objective:** To compare the efficacy of a lottery-based incentive, traditional direct payment incentive, and control of daily feedback without any incentive for weight-loss maintenance<br>**Participants:** Age 30-80, BMI 30-45, who lost at least 5 kg the first 4 months participating in national weight loss program (weight watchers) (n=191)<br>**Country:** United States<br>**Aim of technology:** Weight maintenance<br>**Type of technology or device:** Smartphone, computer<br>**Intervention duration:** 6 months<br>**Blended care:** Yes
**WM6**	Not applicable	**Title:** Design and implementation of an interactive website to support long-term maintenance of weight loss<br>**Authors (year):** Stevens et al. (2008)<br>**Study design:** RCT (4 centers)<br>**Objective:** To describe development and implementation of an maintenance program featuring an Internet website and an associated prompting system using automated email and telephone messages<br>**Participants:** Mean age 56 years, BMI 25-45, taking medication for either hypertension or hyperlipidemia (n=348, that recently lost weight in a 6 month weight loss program)<br>**Country:** United States<br>**Aim of technology:** Weight maintenance<br>**Type of technology or device:** Smartphone, web-based<br>**Intervention duration:** 1 year<br>**Blended care:** No
**WM7**	Not applicable	**Title:** Intervention use and action planning in a web-based computer-tailored weight management program for overweight adults: Randomized controlled trial<br>**Authors (year):** Van Genugten et al. (2014)<br>**Study design:** RCT

		**Objective:** To identify which user characteristics were associated with use of an online, computer-tailored self-regulation intervention aimed at prevention of weight gain; and to examine the quality of the goals and action plans that were generated using the online planning tools.<br>**Participants:** 25-60 years, BMI 25-30 (n= 269)<br>**Country:** The Netherlands<br>**Aim of technology:** Weight maintenance<br>**Type of technology or device:** Computer<br>**Intervention duration:** 8 months<br>**Blended care:** Yes
**WM8**	Not applicable	**Title:** MyPace: An integrative health platform for supporting weight loss and maintenance behaviors<br>**Authors (year):** Barnett et al. (2015)<br>**Study design:** Design paper, prototype described<br>**Objective:** Description of design of myPace, a weight loss and management system via a smartphone and a PC.<br>**Participants:** Dietitians and their patients<br>**Country:** United Kingdom<br>**Aim of technology:** Weight loss & weight maintenance<br>**Type of technology or device:** Smartphone for patients, computer for health personal<br>**Intervention duration:** Not applicable<br>**Blended care:** No
**WM9**	**Weight** ☒<br>**Adherence** ☒<br>**Motivation** ☐	**Title:** Retrofit Weight-Loss Outcomes at 6, 12, and 24 Months and Characteristics of 12-Month High Performers: A Retrospective Analysis<br>**Authors (year):** Painter et al. (2016)<br>**Study design:** Retrospective analysis<br>**Objective:** Evaluation of RETROFIT to assess outcomes related to weight-loss and behavior change<br>**Participants:** Age >18y, BMI >25 (n=2720)<br>**Country:** Canada<br>**Aim of technology:** Weight loss & weight maintenance<br>**Type of technology or device:** Smartphone, web-based<br>**Intervention duration:** 12 months<br>**Blended care:** Yes
**WM10**	Not applicable	**Title:** An evidence-based gamified mHealth intervention for overweight young adults with maladaptive eating habits: Study protocol for a randomized controlled trial<br>**Authors (year):** Podina et al. (2017)<br>**Study design:** Protocol (RCT)<br>**Objective:** The aim of this report is to describe the theoretical rationale and intervention design of the SIGMA study<br>**Participants:** 18-35 years at risk of obesity (BMI 25–29.9 kg/m2) with maladaptive eating habits<br>**Country:** Romania<br>**Aim of technology:** Weight loss & weight maintenance<br>**Type of technology or device:** Smartphone<br>**Intervention duration:** 5 months<br>**Blended care:** No

**WM11**	Not applicable	**Title:** DIABESITY: Design of mHealth integrated solutions for empowering diabetic and obese citizens in self-monitoring and self-management using mobile devices, apps, social media and web-based technologies<br>**Authors (year):** Zoppis et al. (2017)<br>**Study design:** Description of the mHealth platform<br>**Objective:** Description of dietary mHealth tool, explore and measure psychological/behavioral factors to define the type of patient that benefit the most from such an intervention<br>**Participants:** Overweight / obese with diabetes<br>**Country:** Italy<br>**Aim of technology:** Weight loss maintenance<br>**Type of technology or device:** Smartphone<br>**Intervention duration:** Not applicable<br>**Blended care:** Paper describes social networks services prospective for patients and clinicians, and follow-up support of the target group and healthcare personnel.
**WL1**	Not applicable	**Title:** Development of 'Twazon': An Arabic App for Weight Loss<br>**Authors (year):** Alnasser et al. (2016)<br>**Study design:** Design and short pilot-test<br>**Objective:** To describes the process of developing an Arabic weight loss app designed to facilitate the modification of key nutritional and physical activity behaviors among Saudi adults, while taking into consideration cultural norms.<br>**Participants:** Adult, overweight and obese, Saudi women >18y (Saudi health professionals n=5, Saudi women (end users) n=10)<br>**Country:** Saudi-Arabia<br>**Aim of technology:** Weight loss<br>**Type of technology or device:** Smartphone<br>**Intervention duration:** Short pilot<br>**Blended care:** No (only peer support through social network connection possible)
**WL2**	**Weight** ☐<br>**Adherence** ☐<br>**Motivation** ☐	**Title:** The use of mHealth to deliver tailored messages reduces reported energy and fat intake<br>**Authors (year):** Ambeda et al. (2015)<br>**Study design:** 3-armed RCT (single center)<br>**Objective:** To examine the impact of daily feedback messages, delivered remotely, on changes in dietary intake<br>**Participants:** Adults, 18-59 years, BMI 27-43 kg/m2(n=210)<br>**Country:** United States<br>**Aim of technology:** Weight loss<br>**Type of technology or device:** Personal digital assistant<br>**Intervention duration:** 2 years<br>**Blended care:** No
**WL3**	**Weight** ☒<br>**Adherence** ☒<br>**Motivation** ☐	**Title:** Use of a computerized tracking system to monitor and provide feedback on dietary goals for calorie-restricted diets: The POUNDS LOST study<br>**Authors (year):** Anton et al. (2012)<br>**Study design:** RCT (randomization to one of the four diets)<br>**Objective:** To test the efficacy of four macronutrient diets for weight and fat reduction and improvement of health parameters.

		**Participants:** Healthy obese/overweight men and women<br>BMI 25-40 kg/m2, age 30-70 (n=811)<br>**Country:** United States<br>**Aim of technology:** Weight loss<br>**Type of technology or device:** Computer<br>**Intervention duration:** 2 years<br>**Blended care:** Yes
**WL4**	Not applicable	**Title:** An internet-based self-administered intervention for promoting healthy habits and weight loss in hypertensive people who are overweight or obese: a randomized controlled trial<br>**Authors (year):** Banos et al. (2015)<br>**Study design:** Protocol (RCT)<br>**Objective:** Protocol describes a study that aims to assess the efficacy of a totally self-administered online intervention program vs. usual medical care to promote healthy lifestyles (eating behavior and physical activity).<br>**Participants:** Overweight/obese, BMI 25-35, 18 to 65 years, in treatment for prevention of metabolic syndrome or cardiac complications (n=100)<br>**Country:** Spain<br>**Aim of technology:** Weight loss<br>**Type of technology or device:** Computer<br>**Intervention duration:** 3 months<br>**Blended care:** No
**WL5**	**Weight** ☒<br>**Adherence** ☐<br>**Motivation** ☒	**Title:** Text messaging as adjunct to community-based weight management program<br>**Authors (year):** Bouhaidar et al. (2013)<br>**Study design:** A pilot quasi-experimental study with pre-post analysis<br>**Objective:** To evaluate effect of tailored text messages on body weight change in overweight and obese adults (in a community-based weight management program) and to detect behavioral changes.<br>**Participants:** Overweight and obese adults, BMI 25–40 kg/m2, 18 years or older (n=28)<br>**Country:** United States<br>**Aim of technology:** Weight loss<br>**Type of technology or device:** Smartphone<br>**Intervention duration:** 12 weeks<br>**Blended care:** Yes
**WL6**	**Weight** ☒<br>**Adherence** ☒<br>**Motivation** ☐	**Title:** The SMARTER pilot study: Testing feasibility of real-time feedback for dietary self-monitoring<br>**Authors (year):** Bruke et al. (2017)<br>**Study design:** Pilot RCT (3 arms)<br>**Objective:** To test feasibility of providing 1 to 4 daily feedback (FB) messages tailored to dietary recordings via a smartphone and compare the effect of self-monitoring (SM) alone to SM with tailored FB and SM plus tailored FB and face-to-face group sessions.<br>**Participants:** ≥ 18 years, BMI ≥ 27 and ≤43 (n=39)<br>**Country:** United Kingdom<br>**Aim of technology:** Weight loss<br>**Type of technology or device:** Smartphone, computer (App)<br>**Intervention duration:** 12 weeks

		**Blended care:** Yes
**WL7**	**Weight** ☒<br>**Adherence** ☒<br>**Motivation** ☐	**Title:** Adherence to a smartphone application for weight loss compared to website and paper diary: Pilot randomized controlled trial<br>**Authors (year):** Carter et al. (2013)<br>**Study design:** Pilot RCT<br>**Objective:** To test the acceptability and feasibility (recruitment, dropout, and adherence) of My Meal Mate with a view to informing a larger trial.<br>**Participants:** BMI of ≥27 kg/m2; aged 18 to 65 years<br>**Country:** United Kingdom<br>**Aim of technology:** Weight loss<br>**Type of technology or device:** Smartphone<br>**Intervention duration:** 6 months<br>**Blended care:** No
**WL8**	**Weight** ☒<br>**Adherence** ☐<br>**Motivation** ☒	**Title:** Framed, Interactive Theory-Driven Texting: Effects of Message Framing on Health Behavior Change for Weight Loss<br>**Authors (year):** Cohen et al. (2017)<br>**Study design:** 4-arm experimental intervention (feasibility / acceptability evaluation)<br>**Objective:** To ascertain whether participants receiving<br>messages matched to their gain or loss orientation<br>displayed greater motivation to change their eating and exercise behavior, next to feasibility and acceptability testing<br>**Participants:** African American adults, 18 years or older, BMI > 27<br>**Country:** United States<br>**Aim of technology:** Weight loss<br>**Type of technology or device:** Smartphone<br>**Intervention duration:** 28 days<br>**Blended care:** No
**WL9**	**Weight** ☒<br>**Adherence** ☐<br>**Motivation** ☒	**Title:** Theoretical and Behavioral Mediators of a Weight Loss Intervention for Men<br>**Authors (year):** Crane et al. (2016)<br>**Study design:** Data come from a six-month RCT (testing the intervention compared to a waitlist control group)<br>**Objective:** To test the theoretical and behavioral mediators of a men-only internet delivered weight loss intervention<br>**Participants:** Men, 18-65 years old, BMI 25-40 kg/m2 (n=107)<br>**Country:** United States<br>**Aim of technology:** Weight loss<br>**Type of technology or device:** Web-based<br>**Intervention duration:** 6 months<br>**Blended care:** No
**WL10**	Not applicable	**Title:** Track: A randomized controlled trial of a digital health obesity treatment intervention for medically vulnerable primary care patients<br>**Authors (year):** Foley et al. (2016)<br>**Study design:** Protocol (RCT, 2-armed)

		**Objective:** To describe a RCT with aim to test a digital health obesity treatment intervention for medically vulnerable primary care patients<br>**Participants:** Obese men & women, 21-65 years, medically vulnerable with a diagnosis of hypertension, diabetes and/or hyperlipidemia, BMI: 30.0–44.9 kg/m2) (n=351)<br>**Country:** United States<br>**Aim of technology:** Weight loss<br>**Type of technology or device:** Smartphone<br>**Intervention duration:** 12 months<br>**Blended care:** Yes
**WL11**	**Weight** ☒<br>**Adherence** ☐<br>**Motivation** ☐	**Title:** Directive and nondirective e-coach support for weight loss in overweight adults<br>**Authors (year):** Gabriele et al. 2011<br>**Study design:** RCT (3-armed)<br>**Objective:** To examine how different types of e-coach support influence weight loss, behavior change, and engagement in the intervention.<br>**Participants:** BMI 25-40, 30-60 years (n=104).<br>Majority of participants were female (n=87, 83.7%)<br>**Country:** United States<br>**Aim of technology:** Weight loss<br>**Type of technology or device:** Smartphone, computer<br>**Intervention duration:** 12 weeks<br>**Blended care:** Yes
**WL12**	**Weight** ☒<br>**Adherence** ☒<br>**Motivation** ☐	**Title:** A personalized, multi-platform nutrition, exercise, and lifestyle coaching program: A pilot in women<br>**Authors (year):** Heroux et al. 2017<br>**Study design:** Pilot-study, observational design<br>**Objective:** To examine if a personalized web-based multi-platform supported weight loss and the reduction of chronic disease risk factors in overweight or obese women.<br>**Participants:** 18–65 years, men & women, BMI > 24.9 (n=77, 9 men and 68 women)<br>**Country:** Canada<br>**Aim of technology:** Weight loss<br>**Type of technology or device:** Smartphone, computer, or tablet<br>**Intervention duration:** 1 year<br>**Blended care:** Yes
**WL13**	**Weight** ☒<br>**Adherence** ☒<br>**Motivation** ☐	**Title:** Enhancement of self-monitoring in a web-based weight loss program by extra individualized feedback and reminders: Randomized trial<br>**Authors (year):** Hutchesson et al. (2016)<br>**Study design:** RCT<br>**Objective:** To determine whether the consistency of self-monitoring differed between participants randomly assigned to a basic or an enhanced 12 week commercial Web-based weight loss program, and whether the consistency of self-monitoring was related to weight loss<br>**Participants:** Age 18-60, BMI 25-40 (n= 301)<br>**Country:** Australia<br>**Aim of technology:** Weight loss

		**Type of technology or device:** Smartphone, computer<br>**Intervention duration:** 12 weeks<br>**Blended care:** Yes
**WL14**	**Weight** ☒<br>**Adherence** ☐<br>**Motivation** ☐	**Title:** Innovation in weight loss programs: A 3-dimensional virtual-world approach<br>**Authors (year):** Johnston et al. (2012)<br>**Study design:** A comparative study<br>**Objective:** To examine the effectiveness of a virtual world- based weight loss intervention in achieving weight loss, behavioral change, and self-efficacy.<br>**Participants:** Age >18, BMI > 25 (n=54)<br>**Country:** United States<br>**Aim of technology:** Weight loss<br>**Type of technology or device:** Computer<br>**Intervention duration:** 12 weeks<br>**Blended care:** No
**WL15**	**Weight** ☒<br>**Adherence** ☒<br>**Motivation** ☐	**Title:** Patterns of success: Online self-monitoring in a web-based behavioral weight control program<br>**Authors (year):** Krukowski et al. (2013)<br>**Study design:** RCT (part of the trial)<br>**Objective:** To examine patterns of self-monitoring associated with greater weight loss at 6-months<br>**Participants:** BMI 25-50, ≥ 18 years (n=161)<br>**Country:** United States<br>**Aim of technology:** Weight loss<br>**Type of technology or device:** Computer<br>**Intervention duration:** 6 months<br>**Blended care:** Yes
**WL16**	**Weight** ☒<br>**Adherence** ☐<br>**Motivation** ☐	**Title:** Teammates and social influence affect weight loss outcomes in a team-based weight loss competition<br>**Authors (year):** Leahey et al. (2012)<br>**Study design:** Pre-posttest (12 week competition campaign)<br>**Objective:** To investigate the effects of teammates and social influence on individual weight loss during a team-based weight loss competition<br>**Participants:** Overweight/obese adults, BMI≥25 (n=5045)<br>**Country:** United States<br>**Aim of technology:** Weight loss<br>**Type of technology or device:** Online tracking system<br>**Intervention duration:** 12 weeks<br>**Blended care:** No (supported by teammates and social networks / support from friends, family, and coworkers)
**WL17**	Not applicable	**Title:** Healthy weight game!: Lose weight together: The design and evaluation of a serious game for overweight and obesity<br>**Authors (year):** Lentelink et al. (2013)<br>**Study design:** Design/development of the game<br>**Objective:** To investigate the potential of a serious games approach for the prevention and treatment of overweight and obesity<br>**Participants:** Obesity / overweight young adults (18- 25 years) (most highly-educated) (n=53)

		**Country:** The Netherlands<br>**Aim of technology:** Weight loss<br>**Type of technology or device:** Smartphone<br>**Intervention duration:** Not applicable<br>**Blended care:** No
**WL18**	Not applicable	**Title:** Adapting a database of text messages to a mobile-based weight loss program: The case of the middle east<br>**Authors (year):** Massar et al. (2014)<br>**Study design:** (Survey) Design /development of customized database of text messages<br>**Objective:** To present a method to adapt the messaging content of a weight loss application to the context of its users while retaining an effective degree of automation.<br>**Participants:** Overweight and obese, Arabic speaking / natives<br>**Country:** Qatar<br>**Aim of technology:** Weight loss<br>**Type of technology or device:** Smartphone<br>**Intervention duration:** Not applicable<br>**Blended care:** Yes
**WL19**	**Weight** ☒<br>**Adherence** ☒<br>**Motivation** ☐	**Title:** Measurement of self-monitoring web technology acceptance and use in an e-health weight-loss trial<br>**Authors (year):** Ma et al. (2013)<br>**Study design:** RCT (3 arms)<br>**Objective:** To examine participant acceptance and use of a weight and physical activity self-monitoring Web site that was an integral part of two effective lifestyle interventions for weight loss<br>**Participants:** > 18 years, BMI ≥25, prediabetes and / or metabolic syndrome<br>**Country:** United States<br>**Aim of technology:** Weight loss<br>**Type of technology or device:** Web-based, DVD + secure email within electronic health records<br>**Intervention duration:** 15 months<br>**Blended care:** Yes
**WL20**	**Weight** ☒<br>**Adherence** ☒<br>**Motivation** ☐	**Title:** Access to a behavioral weight loss website with or without group sessions increased weight loss in statewide campaign<br>**Authors (year):** Mateo et al. (2014)<br>**Study design:** RCT (3 arms)<br>**Objective:** To determine the efficacy and cost-effectiveness of adding an internet behavioral weight loss intervention alone or combined with optional group sessions to SURI 2011 (a statewide wellness campaign).<br>**Participants:** >18 years, <70 years, BMI > 25 BMI (n=230)<br>**Country:** United States<br>**Aim of technology:** Weight loss<br>**Type of technology or device:** Computer<br>**Intervention duration:** 3 months<br>**Blended care:** Yes
**WL21**	Not applicable	**Title:** Study protocol for the 'HelpMeDoIt!' randomised controlled feasibility trial: An app, web and social support-based weight loss intervention for adults with obesity<br>**Authors (year):** Matthews et al. (2017)

		**Study design:** Protocol (RCT)<br>**Objective:** To test feasibility and acceptability an intervention supporting adults with obesity to achieve weight loss goals<br>**Participants:** Age 18-70, BMI≥30 (n=120)<br>**Country:** Scotland<br>**Aim of technology:** Weight loss<br>**Type of technology or device:** Smartphone, computer<br>**Intervention duration:** 6 months<br>**Blended care:** No (support from one or more helpers within social network (family, friend, colleague))
**WL22**	**Weight** ☒<br>**Adherence** ☒<br>**Motivation** ☐	**Title:** Mobile diary for wellness management - Results on usage and usability in two user studies<br>**Authors (year):** Mattila et al. (2008)<br>**Study design:** Explorative study<br>**Objective:** To study the usage, usability, and acceptance of the weight management implementation of a wellness diary<br>**Participants:** BMI >25, adults, 25-54 years (n=27)<br>**Country:** Finland<br>**Aim of technology:** Weight loss<br>**Type of technology or device:** Smartphone<br>**Intervention duration:** 3 months<br>**Blended care:** Yes
**WL23**	**Weight** ☒<br>**Adherence** ☐<br>**Motivation** ☒	**Title:** Effects of a general practice guided web-based weight reduction program - Results of a cluster-randomized controlled trial<br>**Authors (year):** Mehring et al. (2013)<br>**Study design:** RCT (2 arms)<br>**Objective:** To examine short-term effectiveness of a web-based coaching program in combination with an accompanied telephone counselling regarding weight reduction in a primary care setting<br>**Participants:** Age ≥18, BMI ≥ 25 (n= 186)<br>**Country:** Germany<br>**Aim of technology:** Weight loss<br>**Type of technology or device:** Smartphone, web-based<br>**Intervention duration:** 12 weeks<br>**Blended care:** Yes
**WL24**	Not applicable	**Title:** Face-to-Face and Online Networks: College Students' Experiences in a Weight-Loss Trial<br>**Authors (year):** Merchant et al. (2017)<br>**Study design:** Qualitative study<br>**Objective:** To explore how overweight/obese college students participating in a RCT used social and mobile technologies for weight-related behavior change and leveraged their social networks online and face-to-face while working toward their weight-loss goals.<br>**Participants:** College students (18-24 years), overweight / obese (n=38)<br>**Country:** United States<br>**Aim of technology:** Weight loss<br>**Type of technology or device:** Smartphone

		**Intervention duration:** Not applicable<br>**Blended care:** Yes
**WL25**	Not applicable	**Title:** Optimization of remotely delivered intensive lifestyle treatment for obesity using the Multiphase Optimization Strategy: Opt-IN study protocol<br>**Authors (year):** Pellegrini et al. (2014)<br>**Study design:** Protocol (fractional factorial experimental design)<br>**Objective:** To identify among obese adults which components or component levels contribute meaningfully to improvement in (a) average weight loss, and (b) percent achieving >7% weight loss<br>**Participants:** 18-60 years, BMI 30-40 (n=560)<br>**Country:** United States<br>**Aim of technology:** Weight loss<br>**Type of technology or device:** Smartphone<br>**Intervention duration:** 6 months<br>**Blended care:** Yes
**WL26**	Not applicable	**Title:** Development and feasibility testing of a smart phone based attentive eating intervention<br>**Authors (year):** Robinson et al. (2013)<br>**Study design:** Feasibility testing<br>**Objective:** Describe the development and feasibility of a smartphone trial<br>**Participants:** BMI > 25 who wanted to lose weight, age unknown, (n=12, n=5 overweight + n=7 obese)<br>**Country:** United Kingdom<br>**Aim of technology:** Weight loss<br>**Type of technology or device:** Smartphone<br>**Intervention duration:** 4 weeks<br>**Blended care:** No
**WL27**	**Weight** ☒<br>**Adherence** ☐<br>**Motivation** ☐	**Title:** A comparison of MOVE! versus TeleMOVE programs for weight loss in Veterans with obesity<br>**Authors (year):** Rutledge et al. (2017)<br>**Study design:** Observational comparison study<br>**Objective:** To compare a new telehealth treatment (TeleMOVE) to an established behavioral treatment (MOVE!) among Veterans with obesity.<br>**Participants:** Veterans BMI >30 (n=699, of these 72 from TeleMOVE and 141 from MOVE!)<br>**Country:** United States<br>**Aim of technology:** Weight loss<br>**Type of technology or device:** Telehealth monitor<br>**Intervention duration:** 90 days<br>**Blended care:** Yes
**WL28**	**Weight** ☒<br>**Adherence** ☒<br>**Motivation** ☐	**Title:** Text4Diet: A randomized controlled study using text messaging for weight loss behaviors<br>**Authors (year):** Shapiro et al. (2012)<br>**Study design:** RCT<br>**Objective:** To evaluate a daily text-messaging weight loss intervention<br>**Participants:** Age 21-65, BMI 25-39,9 (n=170)<br>**Country:** United States

		**Aim of technology:** Weight loss<br>**Type of technology or device:** Smartphone<br>**Intervention duration:** 12 months<br>**Blended care:** No
**WL29**	**Weight** ☒<br>**Adherence** ☒<br>**Motivation** ☐	**Title:** An Internet-Based Virtual Coach to Promote Physical Activity Adherence in Overweight Adults: Randomized Controlled Trial<br>**Authors (year):** Watson et al. (2012)<br>**Study design:** RCT (2 arms)<br>**Objective:** To understand the effectiveness of virtual coaching compared with the use of a pedometer and website alone in improving activity levels in overweight or obese participants.<br>**Participants:** BMI 25-35, age 20-55 (n= 70)<br>**Country:** United States<br>**Aim of technology:** Weight loss<br>**Type of technology or device:** Computer<br>**Intervention duration:** 12 weeks<br>**Blended care:** No
**WL30**	**Weight** ☒<br>**Adherence** ☒<br>**Motivation** ☒	**Title:** The effect of a motivational intervention on weight loss is moderated by level of baseline controlled motivation<br>**Authors (year):** Webber et al. (2012)<br>**Study design:** RCT<br>**Objective:** To determine if a motivation-enhanced behavioral weight loss intervention, resulted in greater weight loss, greater program usage and greater increases in autonomous motivation than a standard behavioral weight loss program.<br>**Participants:** Women, age 22-65, BMI 25 -40 (n=80)<br>**Country:** United States<br>**Aim of technology:** Weight loss<br>**Type of technology or device:** Computer<br>**Intervention duration:** 16 weeks<br>**Blended care:** Yes
**WL31**	**Weight** ☒<br>**Adherence** ☒<br>**Motivation** ☒	**Title:** Motivation and Its Relationship to Adherence to Self-monitoring and Weight Loss in a 16-week Internet Behavioral Weight Loss Intervention<br>**Authors (year):** Webber et al. (2010)<br>**Study design:** RCT<br>**Objective:** To examine changes in motivation and the relationship of motivation to adherence to self-monitoring and weight loss.<br>**Participants:** Women, age 22-65, BMI 25 -40 (n=66)<br>**Country:** United States<br>**Aim of technology:** Weight loss<br>**Type of technology or device:** Computer<br>**Intervention duration:** 16 weeks<br>**Blended care:** Yes

**WL32**	**Weight** ☒<br>**Adherence** ☒<br>**Motivation** ☐	**Title:** Using the Habit App for Weight Loss Problem Solving:<br>Development and Feasibility Study<br>**Authors (year):** Pagoto et al. (2018)<br>**Study design:** Two single-arm pilot studies<br>**Objective:** Evaluation of feasibility and acceptability of the Habit app<br>**Participants:** BMI 20 – 45 (n=30)<br>**Country:** USA<br>**Aim of technology:** Weight loss<br>**Type of technology or device:** Smartphone<br>**Intervention duration:** 8 weeks<br>**Blended care:** No
**WL33**	Not applicable	**Title:** The Healthy Hearts and Kidneys (HHK) study: Design of a 2 × 2 RCT of technology-supported self-monitoring and social cognitive theory-based counseling to engage overweight people with diabetes and chronic kidney disease in multiple lifestyle changes<br>**Authors (year):** Sevick et al. (2018)<br>**Study design:** 2x2 factorial RCT<br>**Objective:** Describe the design and methods of the study<br>**Participants:** BMI ≥ 27, age ≥40 with type 2 diabetes and chronic kidney disease<br>**Country:** USA<br>**Aim of technology:** Weight loss<br>**Type of technology or device:** Tablet<br>**Intervention duration:** Not applicable<br>**Blended care:** Yes
**WL34**	**Weight** ☒<br>**Adherence** ☒<br>**Motivation** ☐	**Title:** A Behavioral Lifestyle Intervention Enhanced With<br>Multiple-Behavior Self-Monitoring Using Mobile and Connected Tools for Underserved Individuals With Type 2 Diabetes and Comorbid Overweight or Obesity: Pilot Comparative Effectiveness Trial<br>**Authors (year):** Wang et al. (2018)<br>**Study design:** RCT, 3 arms<br>**Objective:** To examine the feasibility and prelimiary efficacy of a behavioral lifestyle intervention<br>**Participants:** BMI >25, age 21-75 with diabetes mellitus living in underserved communities (n=26)<br>**Country:** USA<br>**Aim of technology:** Weight loss<br>**Type of technology or device:** Smartphone<br>**Intervention duration:** 6 months<br>**Blended care:** Yes
