# Supplementary material for: Persuasive System Design Principles and Behavior Change Techniques to Stimulate Motivation and Adherence in Electronic Health Interventions to Support Weight Loss Maintenance: Scoping Review
Source: J Med Internet Res. 2019 Jun 21;21(6):e14265. doi: 10.2196/14265 (PMC6611151; doi:10.2196/14265)
Supplement: Multimedia Appendix 6 [file jmir_v21i6e14265_app6.pdf]

Multimedia Appendix 6 Reported outcomes*

*Presenting outcomes measured in weight loss interventions >6 months and weight loss maintenance interventions

Study number	Adherence measured / outcomes	Motivation measured / outcomes	Weight measured / outcomes
**WM1**	No	No	No
**WM2**	No	No	No
**WM3**	Yes, adherence to coaching, by sending self-monitoring data to coach, was evaluated. This method cannot be used to evaluate actual usage.<br>Intended usage: >=1/day.	No	Yes, significant effects were found for this intervention. There were significant group differences in weight regain (p=.01).
**WM4**	Yes, adherence measured by the amount of delivered text messages. Adherence to coaching calls and delivery of text messages was reported to be >90%. The method cannot be used to evaluate usage.<br>A separate publication on process evaluation, evaluated use more extensively.<br>Intended usage: >=1/day.	No. Motivation was evaluated in the separate paper, through qualitative methods.<br>Ref: *Process evaluation of TXT2BFiT: a multi-component mHealth randomised controlled trial to prevent weight gain in young adults. Patridge et al. 2016.*	Yes, significant effects were found for this intervention through self-reported measures.
**WM5**	No	No	No
**WM6**	No	No	No
**WM7**	No	No	No
**WM8**	No	No	No
**WM9**	Yes, adherence measured through self-monitoring of weight/weigh-ins and activity tracking. Usage of the dashboard cannot be linked to the actual usage or adherence, or to effective PSD features, as no data on the actual usage is provided. Intended usage: >=1/day.	No	Yes, significant effects were found for this intervention. This intervention has one weight-loss, and one weight maintenance part, and focus on “High- performing participants”= Participants who lost ≥10% of their starting weight.
**WM10**	No	No	No
**WM11**	No	No	No
**WL1**	No	No	No
**WL2**	No	No	No

**WL3**	Yes, indicators of adherence to the prescribed interventions were measured<br>and automatically computed by the computerized tracking system: (1) attendance to individual sessions, (2) attendance to group sessions, (3) frequency of submitting dietary self-monitoring records, (4) frequency of submitting physical activity records, (5) deviation from total daily kilocalories goal, (6) deviation from daily protein goal, (7) deviation from daily fat goal, and (8) deviation from daily carbohydrate<br>goal. Rates of internet based computer tracking system utilization were highest during year 1 of this 2-year intervention, but declined steadily over time.<br>Intended usage: >=1/day.	No	Yes, significant effects were found for this intervention. The key finding was that weight loss from baseline to week 32 was significantly greater for participants with high versus low usage.
**WL4**	No	No	No
**WL5**	No	Yes, participants had to answer questions and telling to what degree they experienced the text messages motivational ("not at all, somewhat, very much")	Yes
**WL6**	Yes, intervention duration ≤6 months.<br>Intended usage: >=1/day	No	Yes
**WL7**	Yes, intervention duration ≤6 months.<br>Intended usage: free usage, not specified	No	Yes

**WL8**	No	Yes, motivational orientation (gain or loss focus) and the focus on behavioral consequences was measured by the instrument Consideration of Future Consequences (CFC) and the Behavioral Inhibition and Activation Scales (BIS/BAS). The Diet and Exercise Self-Efficacy questionnaires and the University of Rhode Island Change Assessment (URICA) scale were also used to assess motivation.	Yes
**WL9**	No	Yes, motivation for healthy eating and continued exercise was assessed by using the autonomous and controlled regulation subscales of the Treatment Self-Regulation Questionnaire, TSRQ.	Yes
**WL10**	No	No	No
**WL11**	No, evaluate engagement	No	Yes
**WL12**	Yes, adherence was measured as reported by the authors, which in this case was compliance to the web-based program. The reported measures related to The Precision Nutrition Coaching Program, a web-based multi-platform, cannot be linked to actual usage, or to effective PSD features.<br>Intended usage: >=1/day.	No	Yes, significant effects were found for this intervention. Completers experienced significant weight loss (between 5 and 10%). Individuals reporting the highest compliance with daily habits and exercise experienced the greatest weight loss.
**WL13**	Yes, intervention duration ≤6 months.<br>Intended usage: >=1/day	No	Yes
**WL14**	No	No	Yes
**WL15**	Yes, intervention duration ≤6 months.<br>Intended usage: >=1/day	No	Yes
**WL16**	No	No	Yes
**WL17**	No	No	No
**WL18**	No	No	No
**WL19**	Yes, adherence was measured according to authors' definition. Web-site usage was according to the authors correlating with weight loss.<br>Intended usage: Every second week (1/month - 1/week).	No	Yes, significant effects were found for this intervention at early stage. The relationship between Heart360 usage and weight loss from 3 to 15 months continued the same trend but was not statistically significant.

**WL20**	Yes, intervention duration ≤6 months. Intended usage: >=1/day	No	Yes
**WL21**	No	No	No
**WL22**	Yes, intervention duration ≤6 months. Intended usage: >=1/day	No	Yes
**WL23**	No	Yes, each week, participants had to report their level of motivation, via an online portal, so that it could be considered whether they needed of closer follow-up from the GP or practice nurse.	Yes
**WL24**	No	No	No
**WL25**	No	No	No
**WL26**	No	No	No
**WL27**	No	No	Yes
**WL28**	Yes, adherence was measured as the total percent of question SMS (knowledge testing, weight and step data) that participants responded to. Participants who responded to a greater proportion of SMS tended to have greater weight loss over time. Intended usage: Step count – daily, weight weekly (1/week).	No	Yes, weight was measured. No group differences in weight loss were found over 6 or 12 months.
**WL29**	Yes, intervention duration ≤6 months. Intended usage: >=1/day	No	Yes
**WL30**	Yes, intervention duration ≤6 months. Intended usage: >=1/day	Yes, weight loss motivation was measured at baseline, at four and sixteen weeks, using the autonomous and controlled regulation subscales of the Treatment Self-Regulation Questionnaire (TSRQ).	Yes
**WL31**	Yes, intervention duration ≤6 months. Intended usage: >=1/day	Yes, motivation for weight loss was measured at baseline and at 4, 8, 12, and 16 weeks using the autonomous and controlled motivation subscales of the Treatment Self-Regulation Questionnaire (TSRQ). Motivation levels were tabulated by averaging the answers for each subscale separately.	Yes

**WL32**	Yes, intervention duration ≤6 months.<br>Intended usage: 2-3/week	No	Yes
**WL33**	No	No	No
**WL34**	Yes, intervention duration ≤6 months.<br>Intended usage: >=1/day	No	Yes

WM = Weight loss maintenance

WL = Weight loss
