# Supplementary material for: Persuasive System Design Principles and Behavior Change Techniques to Stimulate Motivation and Adherence in Electronic Health Interventions to Support Weight Loss Maintenance: Scoping Review
Source: J Med Internet Res. 2019 Jun 21;21(6):e14265. doi: 10.2196/14265 (PMC6611151; doi:10.2196/14265)
Supplement: Multimedia Appendix 7 [file jmir_v21i6e14265_app7.pdf]

Multimedia Appendix 7 Overview over behavior change theories and techniques used in the included eHealth interventions

Study ID	a) Social cognitive theory	b) Cognitive behavioral therapy, ABC model	c) Health Action Process approach model	d) The transtheoretical model	e) Goal setting and action theories	f) Self-regulation theory	g) Regulatory Fit theory	h) Control Theory	i) Self-determination theory	j) Social Support Theories	k) Motivational Interviewing	l) Stroebe´s theory on behavior change	m) Conservation of Resources Theory	n) Michies Behavior Change Wheel	1) Scheduled consequences	2) Reward and threat	3) Repetition and substitution	4) Antecedents	5) Associations	6) Covert Learning	7) Natural consequences	8) Feedback and monitoring	9) Goals and planning	10) Social support	11) Comparison behavior	12) Self-belief	13) Comparison of outcomes	14) Identity	15) Shaping knowledge	16) Regulations	Stimulate adherence	Stimulate motivation
WM1		✓	✓			✓							✓				✓	✓	✓		✓	✓	✓	✓		✓			✓	✓	✓	✓
WM2						✓											✓	✓	✓		✓	✓	✓	✓		✓	✓	✓	✓	✓		✓
WM3																✓			✓			✓	✓	✓					✓		✓	
WM4				✓													✓					✓	✓	✓					✓			✓
WM5																✓			✓			✓	✓								✓	✓
WM6				✓						✓	✓				✓		✓		✓			✓	✓	✓					✓		✓	✓
WM7					✓	✓											✓	✓	✓			✓	✓	✓			✓		✓		✓	✓
WM8											✓											✓	✓	✓							✓	✓
WM9																	✓		✓			✓	✓	✓					✓		✓	
WM10		✓														✓	✓	✓	✓		✓	✓	✓		✓				✓	✓		✓
WM11																	✓					✓	✓	✓					✓		✓	✓
WL1	✓															✓	✓		✓		✓	✓	✓	✓	✓		✓	✓	✓	✓		✓
WL2																						✓	✓						✓			✓
WL3																						✓	✓								✓	
WL4																	✓	✓			✓	✓	✓					✓	✓	✓	✓	

WL 5																	✓					✓	✓								✓	✓
WL 6																						✓	✓						✓			
WL 7																	✓					✓	✓			✓						✓
WL 8							✓										✓					✓	✓	✓					✓			✓
WL 9	✓								✓								✓					✓	✓						✓			✓
WL 10	✓																✓		✓			✓	✓	✓		✓			✓		✓	✓
WL 11																		✓	✓			✓	✓	✓					✓			
WL 12		✓									✓						✓					✓	✓	✓	✓				✓			
WL 13	✓																✓		✓			✓	✓	✓					✓			✓
WL 14	✓																✓	✓	✓			✓	✓	✓	✓			✓	✓			
WL 15						✓											✓					✓	✓	✓		✓		✓	✓			
WL 16	✓															✓			✓			✓	✓	✓	✓						✓	✓
WL 17	✓			✓												✓	✓					✓	✓	✓	✓		✓			✓	✓	✓
WL 18												✓										✓	✓	✓					✓			✓
WL 19																						✓	✓	✓								
WL 20																						✓	✓	✓	✓				✓			
WL 21	✓							✓	✓	✓					✓	✓	✓		✓			✓	✓	✓		✓			✓		✓	✓
WL 22		✓																				✓							✓			✓
WL 23	✓																✓	✓	✓		✓	✓	✓	✓					✓			✓
WL 24																						✓	✓	✓	✓				✓			✓
WL 25								✓									✓	✓	✓			✓	✓	✓					✓		✓	
WL 26														✓			✓	✓	✓			✓	✓						✓			✓
WL 27																	✓		✓		✓	✓	✓	✓					✓			
WL 28	✓																✓	✓				✓	✓						✓		✓	✓
WL 29															✓		✓					✓	✓	✓					✓			✓
WL 30									✓		✓						✓		✓		✓	✓	✓	✓	✓	✓	✓		✓			

WL 31									✓		✓						✓		✓		✓	✓	✓	✓	✓	✓	✓		✓			
WL 32	✓																✓					✓	✓	✓					✓		✓	✓
WL 33																	✓		✓			✓	✓						✓		✓	✓
WL 34						✓											✓		✓		✓	✓	✓	✓					✓		✓	

WM = Weight loss maintenance

WL = Weight loss
