# Supplementary material for: Persuasive System Design Principles and Behavior Change Techniques to Stimulate Motivation and Adherence in Electronic Health Interventions to Support Weight Loss Maintenance: Scoping Review
Source: J Med Internet Res. 2019 Jun 21;21(6):e14265. doi: 10.2196/14265 (PMC6611151; doi:10.2196/14265)
Supplement: Multimedia Appendix 8 [file jmir_v21i6e14265_app8.pdf]

Multimedia Appendix 8 Overview over PSD principles used in the included eHealth interventions

	Primary task support							Dialogue support							Social support							Other				
Study ID	a) Reduction	b) Tunneling	c) Tailoring	d) Personalization	e) Self-monitoring	f) Simulation	g) Rehearsal	h) Praise	i) Rewards	j) Reminders	k) Suggestions	l) Similarity	m) Liking	n) Social role	o) Social learning	p) Social comparison	q) Normative influence	r) Social facilitation	s) Cooperation	t) Competition	u) Recognition	v) Feedback	w) Goal-setting	x) Social support	Stimulate adherence	Stimulate motivation
WM 1			✓	✓	✓	✓		✓		✓	✓											✓	✓		✓	✓
WM 2			✓	✓	✓	✓		✓		✓												✓	✓	✓		✓
WM 3			✓		✓				✓	✓								✓				✓	✓	✓	✓	
WM 4			✓		✓	✓			✓	✓	✓											✓	✓			✓
WM 5			✓		✓				✓	✓												✓	✓		✓	✓
WM 6	✓	✓	✓	✓	✓	✓	✓		✓	✓	✓											✓	✓	✓	✓	✓
WM 7	✓	✓	✓	✓	✓	✓			✓	✓	✓				✓	✓		✓	✓			✓	✓	✓	✓	✓
WM 8			✓	✓	✓	✓		✓		✓	✓											✓	✓	✓	✓	✓
WM 9			✓	✓	✓	✓																✓	✓	✓	✓	
WM 10	✓	✓	✓	✓	✓	✓	✓	✓	✓	✓	✓			✓		✓					✓	✓				✓
WM 11			✓	✓	✓						✓											✓	✓	✓	✓	✓
WL 1	✓	✓	✓	✓	✓	✓	✓	✓	✓	✓	✓		✓		✓	✓	✓		✓			✓	✓	✓		✓
WL 2			✓		✓			✓		✓	✓											✓	✓			✓
WL 3			✓		✓																	✓	✓		✓	
WL 4										✓												✓	✓		✓	
WL 5			✓					✓		✓	✓											✓			✓	✓
WL 6			✓	✓	✓			✓		✓	✓											✓	✓			
WL 7			✓	✓	✓	✓	✓	✓														✓	✓			✓
WL 8			✓							✓	✓											✓				✓
WL 9			✓		✓			✓														✓	✓			✓
WL 10			✓	✓	✓	✓																✓	✓	✓	✓	✓
WL 11			✓	✓	✓	✓				✓												✓	✓			

WL 12	✓			✓	✓	✓	✓	✓		✓	✓				✓	✓						✓	✓	✓		
WL 13			✓	✓	✓					✓												✓	✓	✓		✓
WL 14	✓	✓	✓	✓		✓	✓				✓	✓		✓	✓	✓		✓						✓		
WL 15		✓	✓		✓						✓											✓	✓	✓		
WL 16					✓	✓			✓							✓		✓		✓	✓	✓	✓	✓	✓	✓
WL 17	✓	✓	✓	✓	✓		✓		✓		✓	✓			✓	✓			✓	✓	✓	✓	✓	✓	✓	✓
WL 18			✓		✓			✓		✓	✓								✓			✓	✓	✓		✓
WL 19			✓		✓					✓												✓	✓			
WL 20					✓						✓					✓		✓		✓	✓	✓	✓	✓		
WL 21				✓	✓	✓		✓	✓		✓				✓	✓						✓	✓	✓	✓	✓
WL 22				✓	✓	✓																✓				✓
WL 23			✓		✓					✓	✓											✓	✓	✓		✓
WL 24				✓	✓										✓	✓				✓		✓	✓	✓		✓
WL 25			✓	✓	✓	✓		✓			✓											✓	✓	✓	✓	
WL 26				✓	✓					✓	✓											✓				✓
WL 27					✓																	✓	✓	✓		
WL 28			✓		✓	✓		✓		✓	✓											✓	✓		✓	✓
WL 29			✓	✓	✓	✓		✓			✓			✓								✓	✓	✓		✓
WL 30					✓				✓		✓															
WL 31					✓																			✓		
WL 32		✓	✓	✓	✓	✓				✓	✓				✓							✓	✓	✓	✓	✓
WL 33			✓		✓	✓		✓			✓											✓	✓		✓	
WL 34					✓	✓																✓	✓		✓	

WM = Weight loss maintenance

WL = Weight loss
